# Arylmethylene hydrazine derivatives containing 1,3-dimethylbarbituric moiety as novel urease inhibitors

**DOI:** 10.1038/s41598-021-90104-x

**Published:** 2021-05-19

**Authors:** Keyvan Pedrood, Homa Azizian, Mohammad Nazari Montazer, Maryam Mohammadi‐Khanaposhtani, Mohammad Sadegh Asgari, Mehdi Asadi, Saeed Bahadorikhalili, Hossein Rastegar, Bagher Larijani, Massoud Amanlou, Mohammad Mahdavi

**Affiliations:** 1 Endocrinology and Metabolism Research Center, Endocrinology and Metabolism Clinical Sciences Institute, Tehran University of Medical Sciences, Tehran, Iran; 2 Department of Medicinal Chemistry, School of Pharmacy, Iran University of Medical Sciences, Tehran, Iran; 3 Department of Medicinal Chemistry, Faculty of Pharmacy and Pharmaceutical Sciences Research Center, Tehran University of Medical Sciences, Tehran, Iran; 4 Cellular and Molecular Biology Research Center, Health Research Institute, Babol University of Medical Sciences, Babol, Iran; 5 School of Chemistry, College of Science, University of Tehran, Tehran, Iran; 6 Cosmetic Products Research Center, Iranian Food and Drug Administration, MOHE, Tehran, Iran

**Keywords:** Chemical biology, Computational biology and bioinformatics, Drug discovery, Chemical biology

## Abstract

A new series of arylmethylene hydrazine derivatives bearing 1,3-dimethylbarbituric moiety **7a–o** were designed, synthesized, and evaluated for their in vitro urease inhibitory activity. All the title compounds displayed high anti-urease activity, with IC_50_ values in the range of 0.61 ± 0.06–4.56 ± 0.18 µM as compared to the two standard inhibitors hydroxyurea (IC_50_ = 100 ± 0.15 μM) and thiourea (IC_50_ = 23 ± 1.7 μM). Among the synthesized compounds, compound **7h** with 2-nitro benzylidene group was found to be the most potent compound. Kinetic study of this compound revealed that it is a mix-mode inhibitor against urease. Evaluation of the interaction modes of the synthesized compounds in urease active site by molecular modeling revealed that that compounds with higher urease inhibitor activity (**7h**, **7m, 7c, 7l**, **7i**, and **7o,** with IC_50_ of 0.61, 0.86, 1.2, 1.34, 1.33, 1.94 μM, respectively) could interact with higher number of residues, specially Arg609, Cys592 (as part of urease active site flap) and showed higher computed free energy, while compounds with lower urease activity (**7f**, **7n**, **7g**, and **7a** with IC_50_ of 3.56, 4.56, 3.62 and 4.43 μM, respectively) and could not provide the proper interaction with Arg609, and Cys592 as the key interacting residues along with lower free binding energy. MD investigation revealed compound **7h** interacted with Arg609 and Cys592 which are of the key residues at the root part of mobile flap covering the active site. Interacting with the mentioned residue for a significant amount of time, affects the flexibility of the mobile flap covering the active site and causes inhibition of the ureolytic activity. Furthermore, in silico physico-chemical study of compounds **7a–o** predicted that all these compounds are drug-likeness with considerable orally availability.

## Introduction

*Helicobacter pylori* (*H. pylori*) is one of the bacteria that has caused many problems for humans by lowering their quality of life^1^. This bacteria in addition to causing a variety of gastrointestinal disorders, can even cause gastric cancer^2^. A most common treatment for inhibition of colonization of *H. pylori* is use of a triple therapy containing a proton pump inhibitor and two antibiotic agents^3^. The use of this treatment and similar treatments, in addition to causing side effects in other organs of the body, has led to *H. pylori* antibiotic resistance^4^. Therefore, the use of methods that specifically attack to bacteria is very valuable in the treatment of this disease^5^. One of the most popular of these methods is the use of *H. pylori* urease inhibitors^6^. *H. pylori*, like many other microorganisms, uses urea for growth, thus, the enzyme that breaks down urea, urease, plays an important role in its survival^7^. Several urease inhibitors with various structures have been introduced that can be useful for treatment of *H. pylori* infection^8–10^.

**Figure 1:**
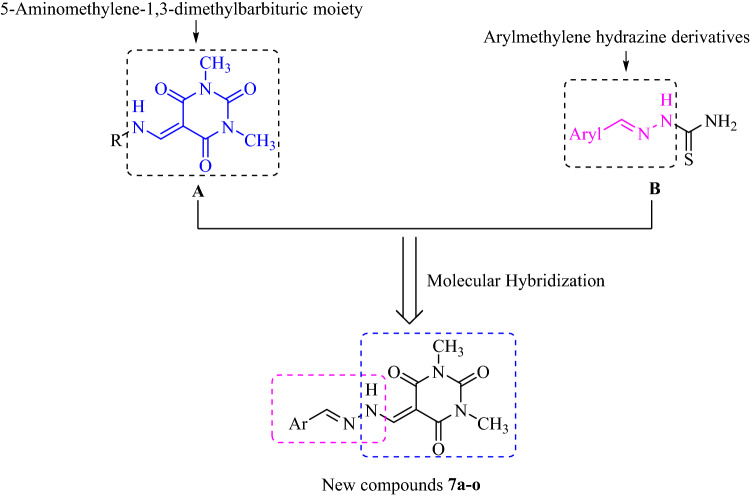
Design strategy for new arylmethylene hydrazine-1,3-dimethylbarbituric derivatives as urease inhibitors.

Recently, barbituric acid and its derivatives such as 1,3-dimethylbarbituric and thiobarbituric have received increasing attention in the discovery of new urease inhibitors^11–13^. Several compounds possessing barbituric acid derivatives such as compounds **A** display high inhibitory activity against urease (Fig. 1)^14^. As can be seen in Fig. 1, the later compounds have a 5-aminomethylene-1,3-dimethylbarbituric moiety. On the other hand, Saeed et al. reported the synthesis of a series of arylmethylene hydrazine derivatives containing carbothioamide **B** as novel urease inhibitors^15^. Their biological data demonstrated that the most potent compound among the compounds **B** showing up to 36.2-fold higher inhibitory potency than standard inhibitor thiourea (Fig. 1). By considering the potent urease inhibitors **A** and **B**, in continuing our efforts to synthesize new urease inhibitors using simple chemical reactions, here, a new series of urease inhibitors **7a–o** was designed by combination of 5-aminomethylene-1,3-dimethylbarbituric moiety and arylmethylene hydrazine derivatives (Fig. 1)^16–20^. Compounds **7a–o** were synthesized by a simple three-step procedure. All these compounds were evaluated for their in vitro urease inhibitory activities. Furthermore, molecular modeling and molecular dynamic studies of compounds **7a–o** were also performed.

## Results and discussion

### Chemistry

**Figure 2:**
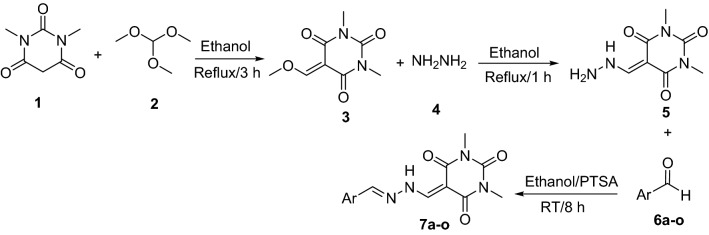
Synthesis of arylmethylene hydrazine-1,3-dimethylbarbituric derivatives **7a–o**.

Arylmethylene hydrazine-1,3-dimethylbarbituric derivatives **7a–o** were synthesized by described method in Fig. 2. According to this method, reaction of 1,3-dimethylbarbituric acid **1** and trimethoxymethane **2** in ethanol under reflux condition afforded 5-(methoxymethylene)-1,3-dimethylbarbituric acid **3**. The latter compound was reacted with hydrazine **4** in ethanol under reflux for produce 5-(hydrazineylmethylene)-1,3-dimethylbarbituric acid **5**. In the final step, compound **5** and aromatic aldehydes **6a–o** in the presence of catalytic amount of PTSA in ethanol at room temperature were converted to desired compounds **7a–o**.

### Urease inhibition

The effect of arylidenehydrazineyl-1,3-dimethylbarbituric derivatives **7a–o** on urease was determined using *Jack bean* (*JB*) urease in accordance to the reported protocols^18–20^. The obtained IC_50_ values of title compounds against urease were presented in Table 1, in comparison with hydroxyurea and thiourea as reference inhibitors.

**Table 1:** The IC_50_ values (μM) of compounds **7a–o** against urease.


Compound	R	IC_50_ (μM)^a^
**7a**	Phenyl	4.43 ± 0.21
**7b**	2-Hydroxyphenyl	3.09 ± 0.13
**7c**	2,4-Hydroxyphenyl	1.2 ± 0.05
**7d**	3,4,5-Trimethoxyphenyl	2.53 ± 0.11
**7e**	3-Phenoxyphenyl	2.85 ± 0.23
**7f**	4-Chlorophenyl	3.56 ± 0.16
**7g**	3-Boromophenyl	3.62 ± 0.19
**7h**	2-Nitrophenyl	0.61 ± 0.06
**7i**	4-Nitrophenyl	1.31 ± 0.09
**7j**	2,3-Dichlorophenyl	4.52 ± 0.31
**7k**	2-Chloro-5-nitrophenyl	3.94 ± 0.25
**7l**	2-Nitro-5-chlorophenyl	1.34 ± 0.12
**7m**	Thiophen-2-yl	0.86 ± 0.08
**7n**	5-Chlorothiophen-2-yl	4.56 ± 0.18
**7o**	Naphthalen-1-yl	1.94 ± 0.22
Hydroxyurea	–	100 ± 0.15
Thiourea	–	23 ± 1.7

^a^Values are the mean ± standard error of the mean. All experiments were performed at least three times.

Generally, all newly synthesized compounds **7a–o**, with IC_50_ values in the range of 0.61 ± 0.06–4.56 ± 0.18 μM, had significant inhibitory effect against urease. All these compounds were more potent than that of hydroxyurea (IC_50_ = 100 ± 0.15 μM) and thiourea (IC_50_ = 23 ± 1.7 μM). The 2-nitrophenyl derivative **7h** and thiophen-2-yl derivative **7m** with IC_50_ values of 0.61 ± 0.06 and 0.86 ± 0.08 μM were found to be the most active compounds. In particular, compound **7h** was 37.7 times more potent than strong urease inhibitor thiourea.

Un-substituted phenyl derivative **7a**, and 2,3-dichlorophenyl derivative **7j**, were weaker than other phenyl derivatives against urease. Among the phenyl derivatives, the most potent compound was 2-nitro derivative **7h**. The activity of this compound was 7.2-fold superior to that of parent phenyl derivative **7a**. Movement of nitro substituent of 2-position into 4-position, as in compound **7i**, and or replacement of 2-nitro group with 2-hydroxy substituent, as in compound **7b**, decreased inhibitory activity to about two and fivefold, respectively. Moreover, introduction of 2,3-dichloro (compound **7j**), 2-chloro-5-nitrophenyl (compound **7k**), 3-bromo (compound **7g**), 4-chloro (compound **7f**), 3-phenoxy (compound **7e**), and 3,4,5-trimethoxyphenyl (compound **7d**) substituents instead of 2-nitro substituent dramatically decreased anti-urease activity while presence of 2,4-hydroxy (compound **7c**) and 2-nitro-5-chloro (compound **7l**) substituents on phenyl ring reduced inhibitory activity with less intensity in comparison to 2-nitro substituent.

The second most potent compound among the synthesized compound was thiophen-2-yl derivative **7m**. As can be seen in Table 1, introduction of 5-chloro substituent on thiophen ring, as in compound **7n**, led to a significant decrease in inhibitory activity. Furthermore, naphthalen-1-yl derivative **7o** was one of the strongest compounds among the synthesized compounds.

### Urease kinetic study

**Figure 3:**
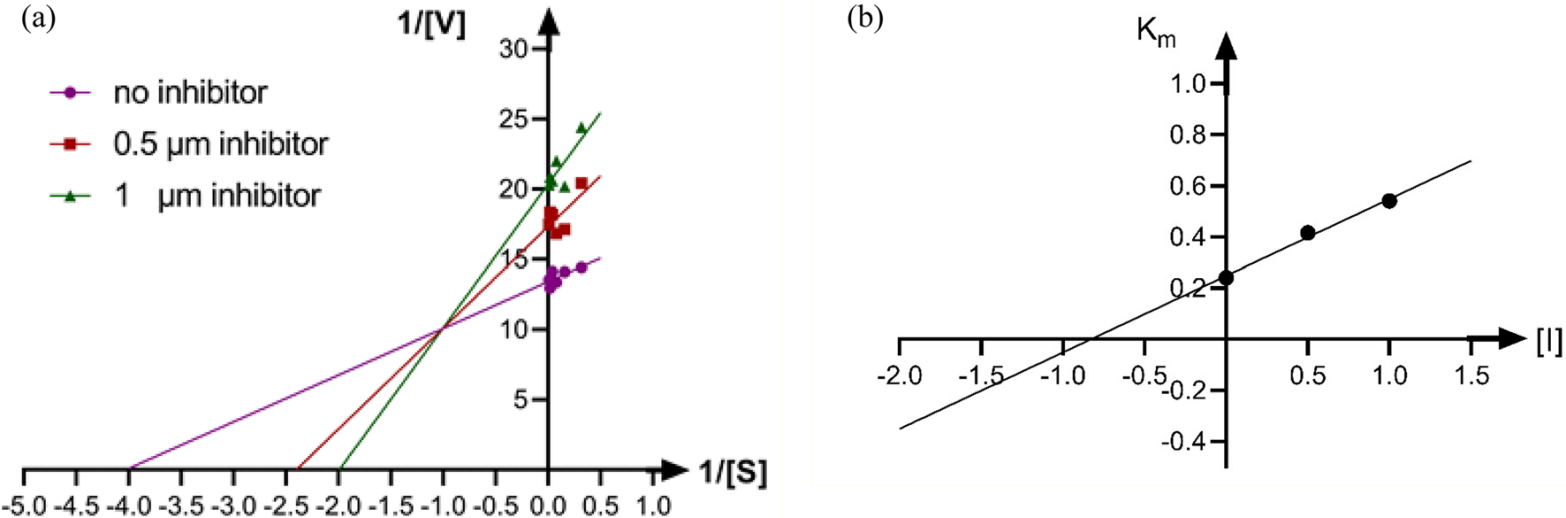
Kinetic analysis of urease inhibition by compound **7h**. (**a**) The Lineweaver–Burk plot in the absence and presence of different concentrations of compound **7h**; (**b**) The secondary plot between 1/V_max_ and various concentrations of compound **7h**.

To evaluate the mechanism inhibition of new arylmethylene hydrazine-1,3-dimethylbarbituric derivatives, kinetics study was performed on the most potent urease inhibitor **7h** (Fig. 3). The inhibition mode and K_i_ value were determined by Lineweaver–Burk plots and secondary re-plotting of the mentioned plots, respectively. As exhibited in Fig. 3a, with increasing concentrations of compound **7h**, V_max_ and K_m_ increased. Therefore, this compound was a mixed-type inhibitor for urease (Fig. 3b, K_i_ = 0.82 µM).

### Docking study

Docking study was applied to distinguish interactions between the synthesized compounds and urease active site. The reliability of the applied docking protocol was assessed according to our previous study by re-docking of acetohydroxamic acid (AHA) into the active site of the *JB* urease^20^. This protocol was then similarly applied to all the synthesized compounds **7a–o**.

The interaction mode of the top IFD scoring of all the synthesized compounds (**7a–o**) showed they successfully occupied in the bi-nickel active site cavity.

**Figure 4:**
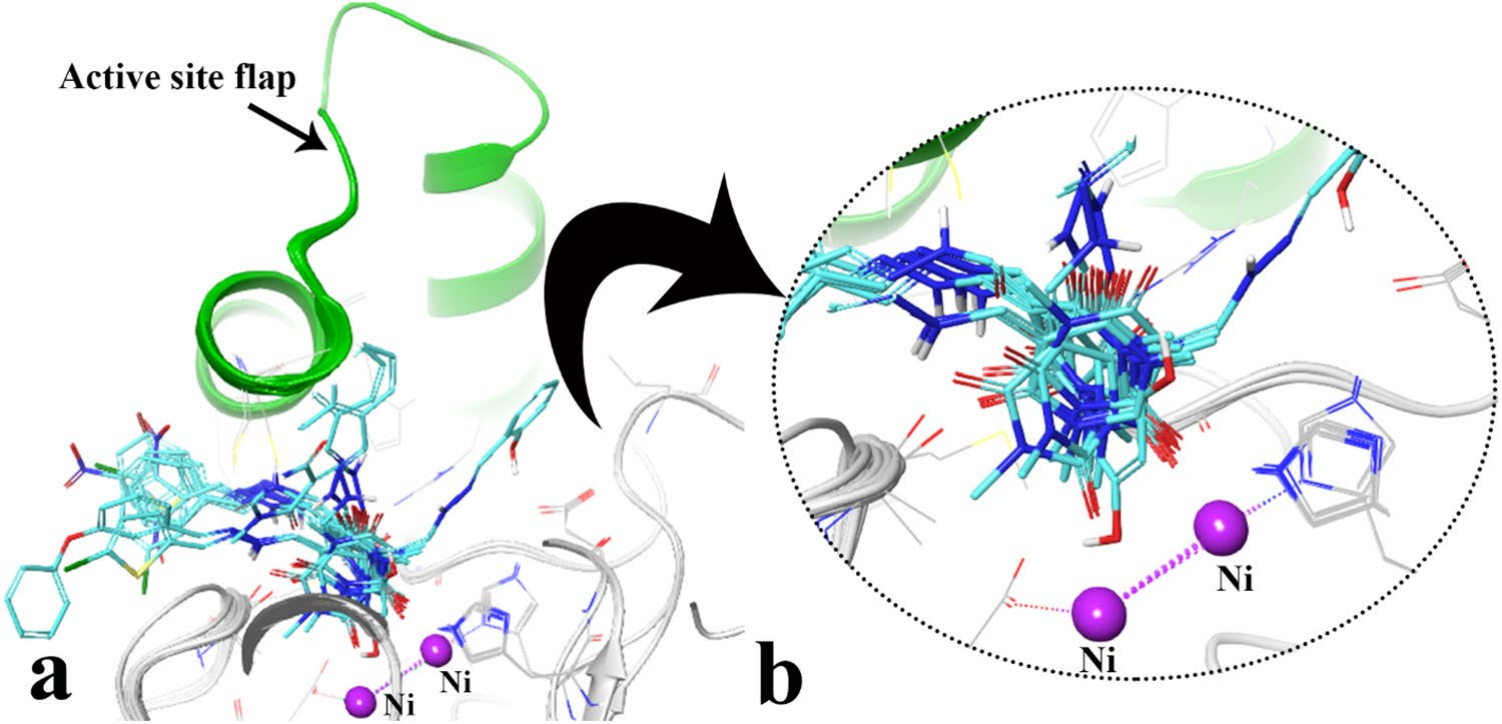
Representation of the compounds docking poses over the active site (**a**) close-up illustration of barbiturate ring relative to the bi-nuclear center (**b**), the active site flap colored in green color. The molecular graphic in this figure was generated using VMD 1.9.3.

Figure 4a shows the top IFD pose of the compounds over *JB* urease. The result depicts superimposing of barbituric acid ring of all the compounds pointed toward the bi-nickel center atoms through the carbonyl group at C_2_ position of the barbiturate ring (Fig. 4b). The orientation of the mentioned carbonyl group is the same as the carbonyl oxygen in the AHA and thiourea as reference inhibitors. Furthermore, the arylmethylene hydrazine moieties oriented to the entering site of the active site and adapted by flexible conformation in the large hydrophobic opening of the active site flap pocket (Fig. 4a). The mentioned extended moiety in designing of the synthesized compounds has the superior inhibitory effect in comparison to the standard inhibitor (thiourea) through higher stabilization effect by implementing of various non-bonding interactions.

**Figure 5:**
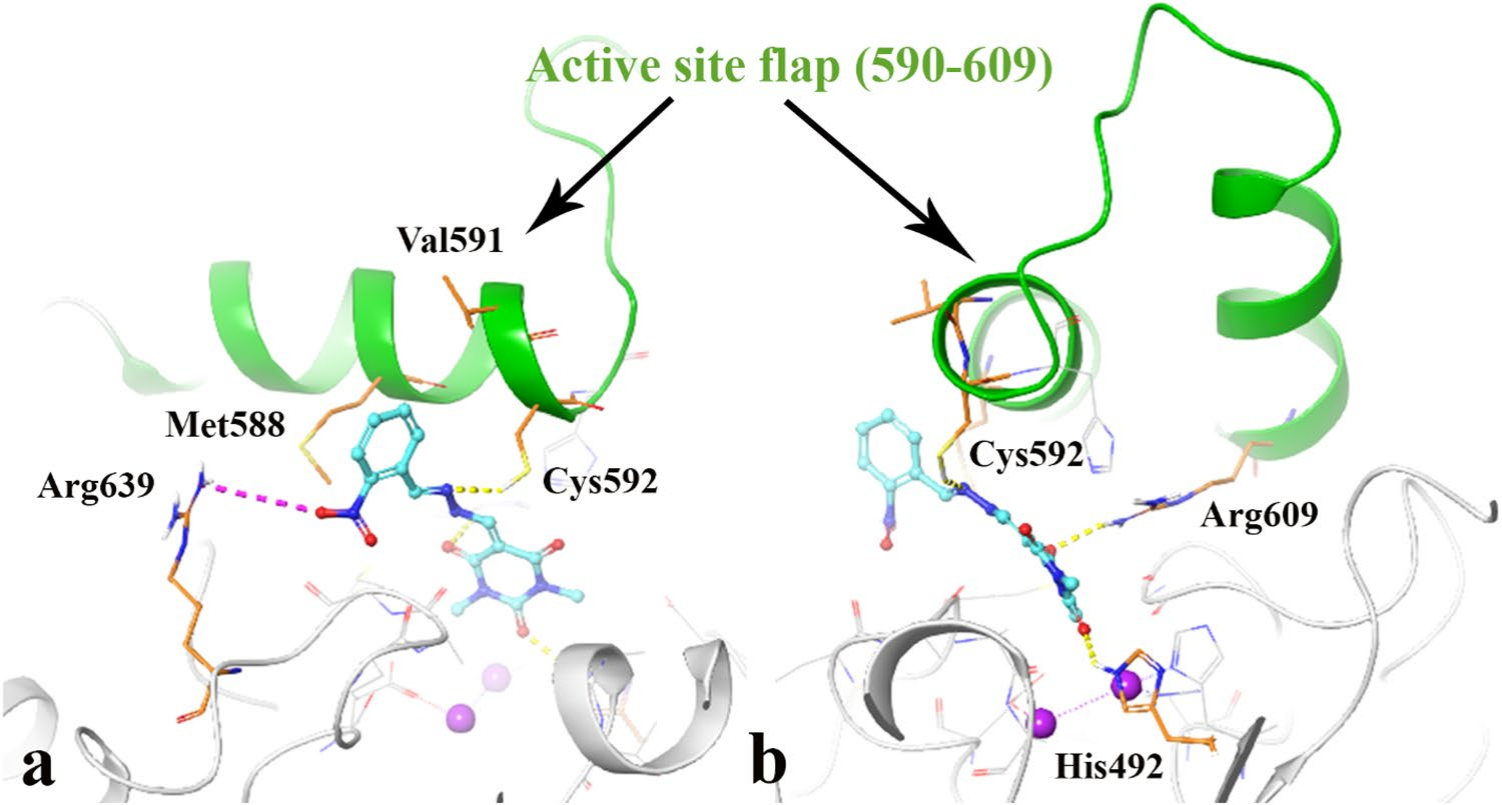
Close up representation of the best energy value of the most active compound **7h** complexed with *JB* urease (**a**), 90-degree anti-clockwise rotation view (**b**). The molecular graphic in this figure was generated using VMD 1.9.3.

In case of the most active compound **7h** (Fig. 5), the carboxyl groups in C_2_ and C_4_ position of barbituric acid ring created H-bond with His492 and Arg609, the hydrazone group formed H-bond interaction through Cys592 (SH) and the ortho nitro benzylidene group was able to form salt-bridge interaction toward Arg639 (Fig. 5a). As a result, compound **7h** well occupied the active pocket of urease and tightly anchoring the helix-turn-helix motif through interacting with Cys592 and Arg609 over the active-site cavity (depicted in green cartoon) (Fig. 5b), which could reduce the flexibility of flap residue (590–609) and results in the inhibition of urease activity.

In order to reveal the effect of different aromatic ring instead of phenyl ring, the docked pose complex and the gibbs free binding energy (ΔG) of the compounds **7a**, **7m** and **7o** were compare to each other.

**Figure 6:**
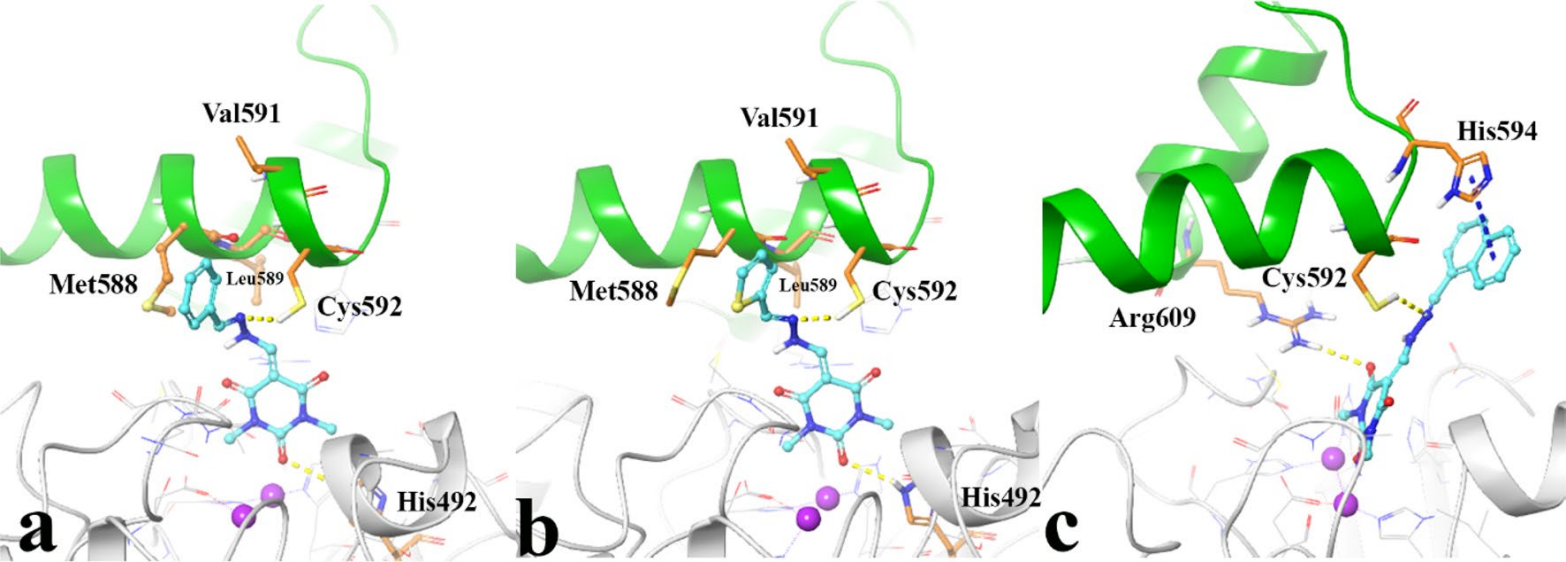
3D representation of ligand-residue interactions of compound **7a** (**a**), compound **7m** (**b**), compound **7o** (**c**) over urease active site. Active site flap colored in green. The molecular graphic in this figure was generated using VMD 1.9.3.

Figure 6a shows barbituric acid ring of compound **7a** tightly coordinated along the metal bi-nickel center and further stabilized by H-bond interaction with His492. At the middle part of the molecule, the hydrazone group formed H-bond interaction through Cys592 (SH). In addition, the benzylidene group at the tail part of the compound formed hydrophobic interaction with hydrophobic pocket formed by Cys592, Met588, Val 591 and Leu589.

Like compound **7a**, compounds **7m** and **7o**, which have different aromatic moieties (thiophenyl and naphthyl, respectively), formed similar H-bond interactions through their barbituric acid ring and hydrazone group with His492 and Cys592, respectively. The gibbs free binding energy indicates higher value (ΔG) of − 46.47 and − 44.27, respectively as compared to compound **7a** with ΔG value of − 39.52 kcal mol^−1^ (Table 2), which is in accordance with the experimental urease activity results (0.86, 1.94 and 4.43 μM, respectively, Table 1). In the case of compound **7m**, the sulfur atom increased hydrophobic interaction by interacting with the mentioned hydrophobic pocket through sulfur atom of Met388, while in the case of compound **7o**, the naphthyl ring rotated as a result of bigger size and interacted with the imidazole ring of His594 by π–π stacking interaction (Fig. 6b,c).

**Table 2:**
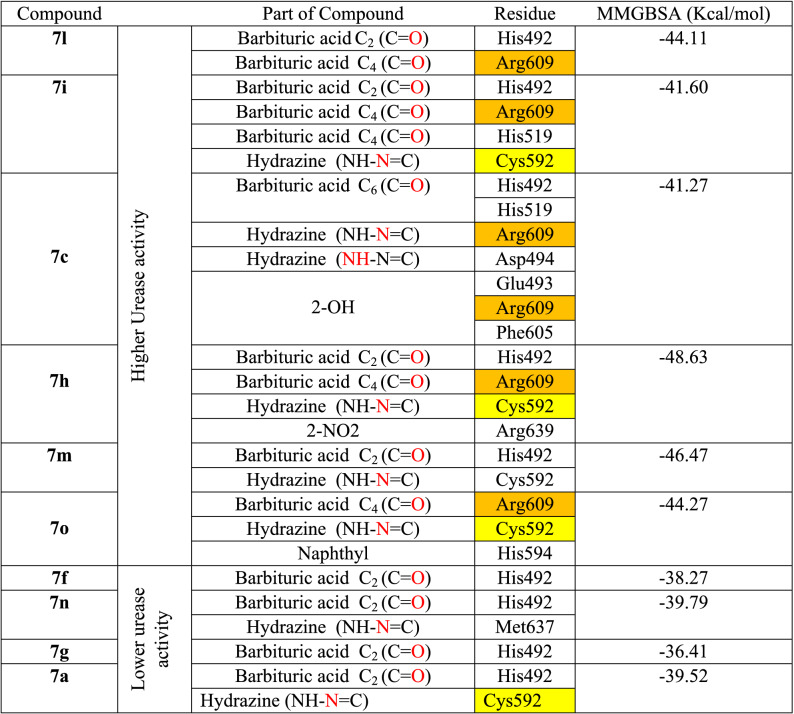
The calculated gibbs free binding energy and the residue involvement in IFD complex of some of the synthesized compounds.

The result shows that the bioisoester substitution of phenyl group provides additional interactions for the compounds **7m** and **7o** to bind to the hydrophobic part of urease active site pocket.

This study confirms our previous investigation which outstands the key role of Cys592 and Arg609 in flexibility of mobile flap covering the active site entrance which consequently affect the inhibition activity of urease enzyme^21^.

Table 2 shows the interaction part of the molecule, the involving residues and the calculated gibbs free binding energies of the compound with higher and lower urease inhibition activity over *JB* urease active site. It indicates that compounds with higher urease inhibitor activity (**7h**, **7m, 7c, 7l**, **7i**, and **7o,** with IC_50_ of 0.61, 0.86, 1.2, 1.34, 1.33, 1.94 μM, respectively) could interact with higher number of residues, specially Arg609, Cys592 (as part of urease active site flap), while compounds with lower urease activity (**7f**, **7n**, **7g**, and **7a** with IC_50_ of 3.56, 4.56, 3.62 and 4.43 μM, respectively) have insufficient interaction and could not provide the proper interaction with Arg609, and Cys592 as the key interacting residues. Furthermore, this finding additionally supported by comparing the gibbs free binding energy of the mentioned compounds which are in accordance with the experimental urease inhibition activity.

### Conformational analyses of the synthesized compounds over the urease active site

**Figure 7:**
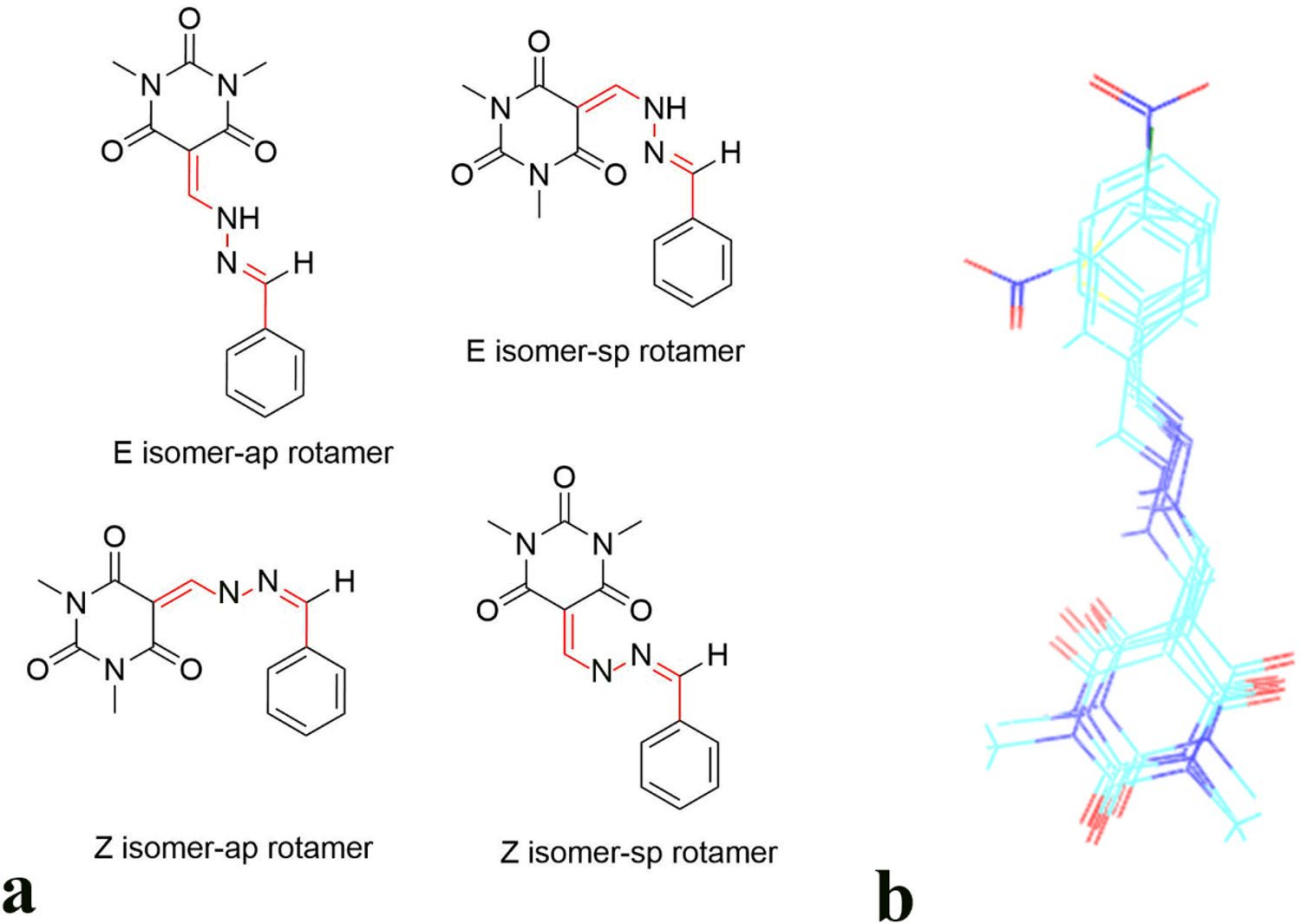
Four possible configurations of benzylidene hydrazone moiety of the synthesized compounds (**a**). Superimposition of the docked structures in the active site of urease (**b**). The 2D structure representation was drawn by ChemAxon Marvin 15.10.12.0^30^.

As the synthesized compounds hold benzylidene hydrazone moiety, they may adopt in four possible geometrical configurations including; the anti-periplanar (ap) and syn-planar rotameric (sp) forms for each Z and E isomers as shown in Fig. 7a. The superimposed structures of IFD compounds over the active site of *JB* urease have been shown in Fig. 7b. It represents that n-π conjugation result in a planar form of C=N–NH moiety. Considering Fig. 7a isomer E with anti-periplanar rotamer was the only configuration while no docked-structures got the Z configuration around C=N bond. This result is in accordance well with a previously reported study where there is no Z configuration around C=N of benzylidene hydrazone moiety due to the steric hindrance^22–24^. Therefore, according to the result it reveals that the E-ap form is the preferred geometrical isomer by the benzylidene hydrazone structure of barbituric acid derivatives.

### Molecular dynamic (MD) investigation

MD study has been investigated in order to reveal the effect of the most potent compound (compound **7h**) on the urease structure and the active site environment in comparison to thiourea as the urease standard inhibitor.

**Figure 8:**
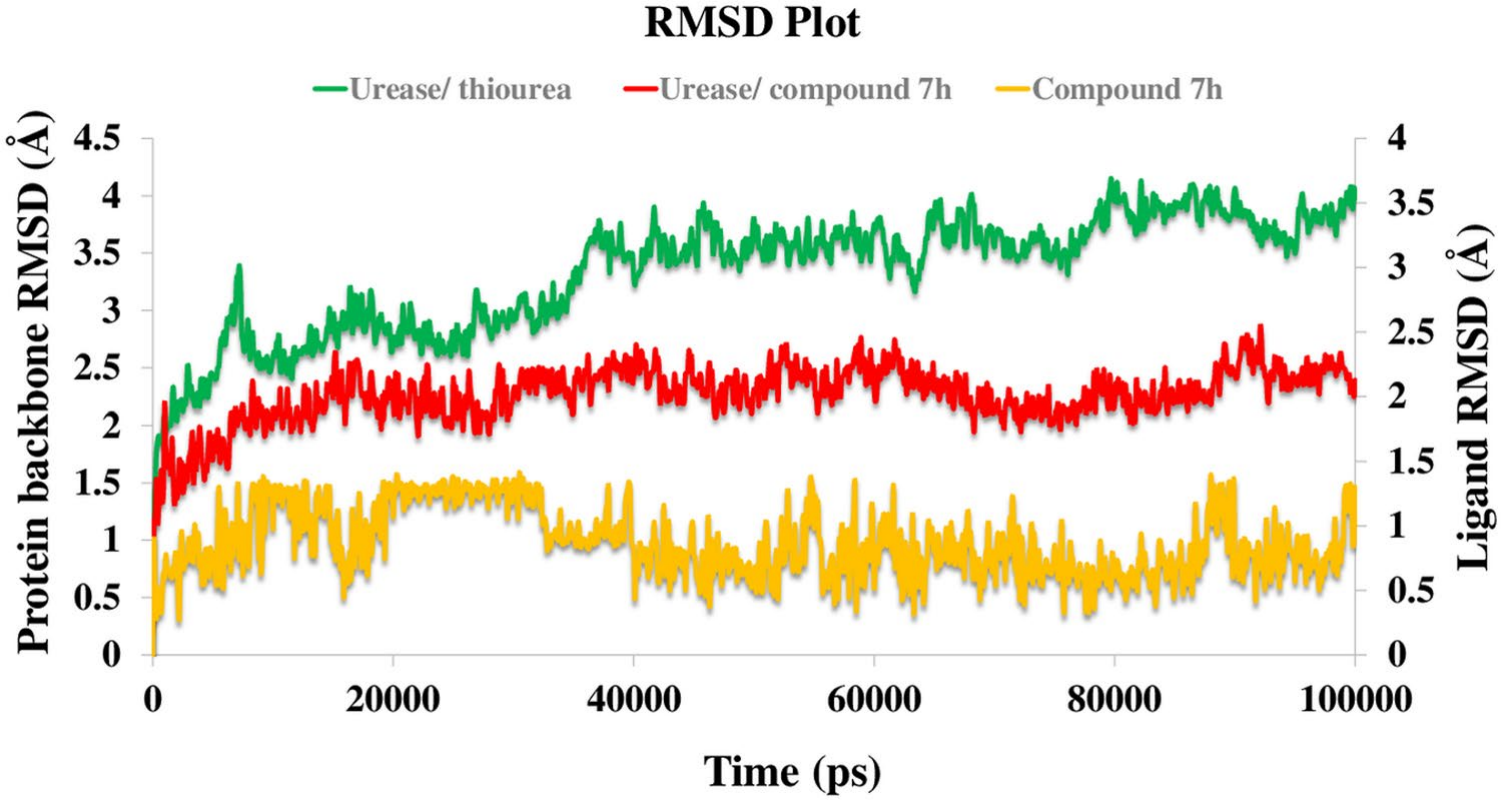
RMSD representation of the compound **7h** (in yellow) and urease backbone in complex with thiourea (in green) and in complex with compound **7h** (in red) for over 100 ns MD simulation time. The plot in was generated using Microsoft Excel (https://www.office.com/).

Figure 8 shows the RMSD of the protein’s backbone over 100 ns MD simulation time. The RMSD simulation showed urease complexed with thiourea maintained an overall stability after 35 ns of MD simulation time with higher RMSD stabilizing at an average of 3.80 Å (Fig. 8, green line), while urease bound-state with compound **7h** displayed longer equilibration time (after 15 ns of MD simulation) with obviously lower RMSD (2.4 Å) (Fig. 8, red line). In addition, the backbone RMSD of compound **7h** equilibrated after about 40 ns and stabilized through the rest of the simulation time with a low RMSD fluctuation around 1.1 Å. Based on the RMSD result it is revealed that the urease-compound **7h** complex obtain an equilibrium structure over the simulation time which has enough stability to investigate the structural specificity of the ligand–protein complex.

**Figure 9:**
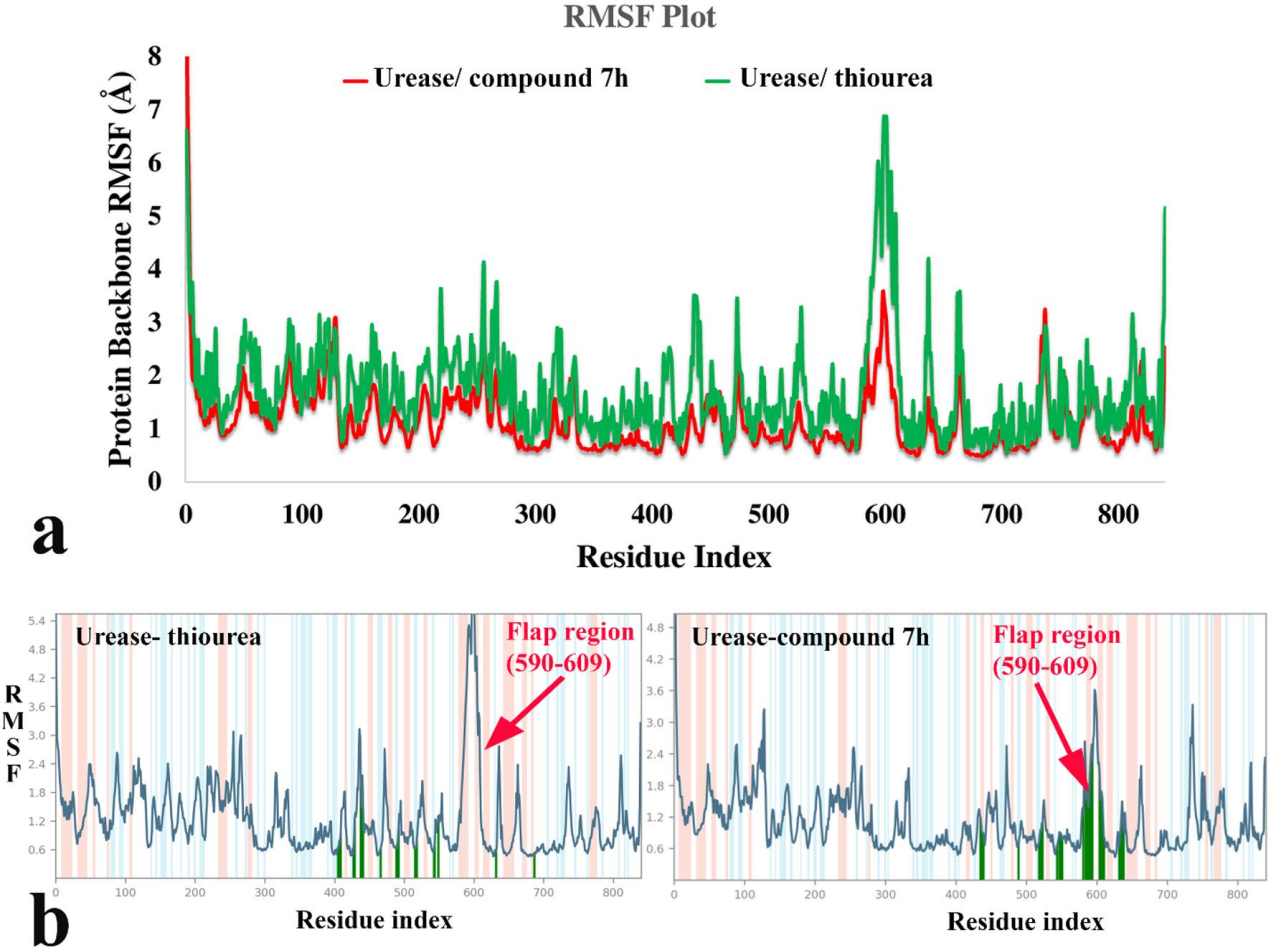
RMSF of the urease backbone in complexed with thiourea (in green) and compound **7h** (in red) (**a**), ligand binding location for over 100 ns MD simulation time; (**b**) α-helical and ß-strand regions are highlighted in light red and blue backgrounds, respectively. The molecular graphic in this figure was generated using VMD 1.9.3.

RMSF define as the fluctuation of the protein’s residues from its average position throughout the simulation, which represents the flexibility of protein structure. In this manner, helixes and sheets with organized structure depict lower RMSF value while loops with loosely organized structure have high RMSF value. Comparing RMSF values of urease-compound **7h** complex shows that it has obviously lower value through the whole part of the urease structure and specially the residues 590–606 showed significantly decreased RMSF value in the urease bound-state with compound **7h** than urease-thiourea complex (Fig. 9a). The mentioned conserved residues with helix-turn-helix secondary structure belong to the α-subunit part of the enzyme known as mobile flap region covering the urease active site^25^.

Figure 9b shows that compound **7h** well occupied the active pockets of urease and tightly anchoring the helix-turn-helix motif over the active-site cavity (vertical green line), which considerably reduce the flexibility of the mobile flap residue (590–609) by interacting with key amino acid residues and results in the inhibition of urease activity. The mentioned interactions could not be observed for the urease-thiourea complex which proposed the role of rigidity of the mobile flap in higher urease inhibition activity of the synthesized compounds.

**Figure 10:**
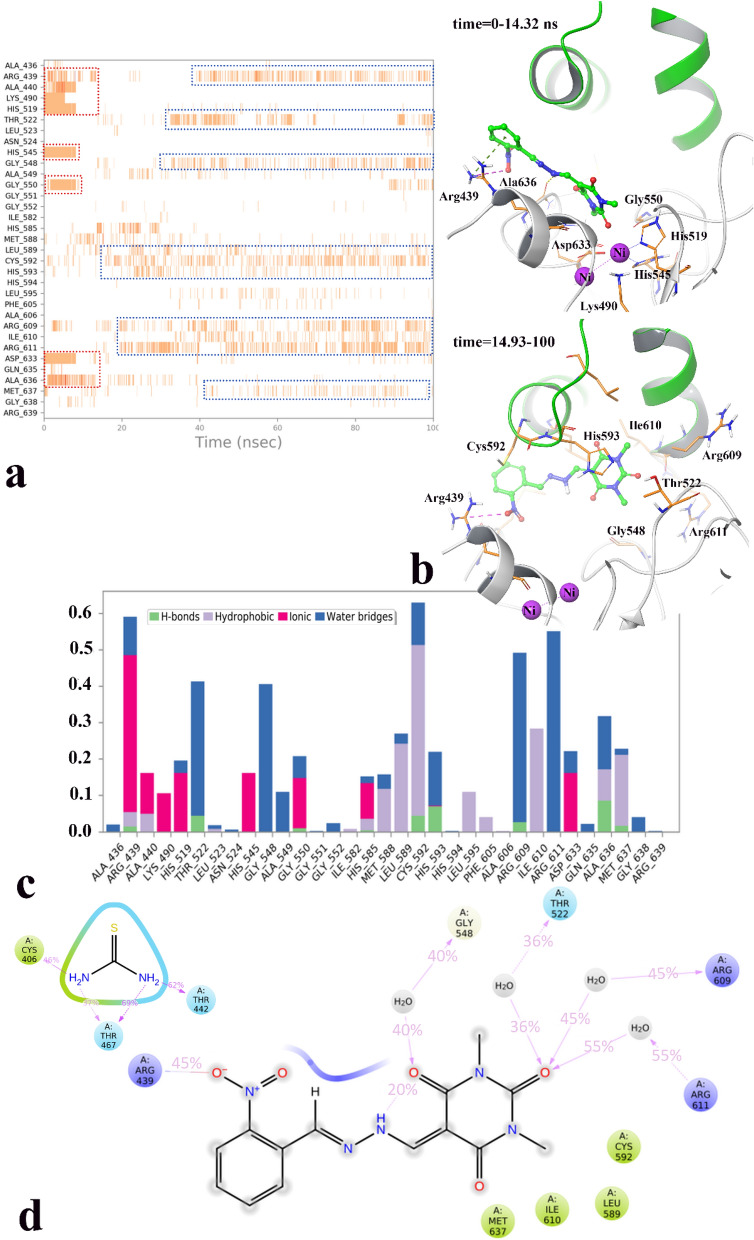
The timeline representation of the interactions shows the residues interact with compound **7h** in each trajectory frame (more than one specific contact with the ligand is represented by a darker shade of orange) (**a**). The 3D representation of urease in bound-state with compound **7h** in two different orientations related to 0–14.32 ns and 14.32 to the rest of simulation time (active site flap are depicted in green cartoon mode) (**b**). The simulation interactions diagram panel. The stacked bar charts are normalized over the course of the trajectory: some protein residues may make multiple contacts with the ligand (**c**). 2D representation of ligand-residue interactions that occur at least 30% of simulation time of urease bound-state of thiurea and compound **7h** (**d**). The molecular graphic in this figure was generated using VMD 1.9.3.

Figure 10 represents different residues, types of interactions, and ligands functional group involvement during the whole MD simulation time.

Based on result, compound **7h** interacted with Arg439, Ala440, Lys490, His519, His545, Gly550, Asp633 and Ala636 for approximate the first 10 ns of the MD simulation (Fig. 10a, red dash boxes). Otherwise, after about 14.32 ns until to the end of simulation the metal-coordinated residue interactions disappeared and substituted with residues; Thr522, Gly548, Leu589, Cys592, His593, Arg609, Arg611, Ile610, and Met637 (Fig. 10a, blue dash boxes).

So, because of the shifting orientation of compound **7h** as a result of different interacting residues, the MD simulation time divided in two sections; 0 ns to 14.32 ns and 14.32 ns to 100 ns. Based on the cluster analysis of compound **7h**, the percent of population in cluster 1 was 86.37% in the first section and 95% in the second section in which the representative frame from cluster 1 of the section 1 (Fig. 10b, up) and the section 2 (Fig. 10b, down) were selected for investigating the 3D complex interaction.

As it is obvious in Fig. 10b (up), at the first stage of MD compound **7h** tightly coordinated toward the active site bi-nickel center through its barbituric acid. In addition, the ortho nitro benzylidene moiety provided ion-bridge and π-cation interactions through Arg439. Otherwise, after about 14 ns the barbituric acid ring shifted from the bi-nickel center toward the active site flap and provided interactions with Cys592, His593 at one side and Arg609 and Arg611 at the other side of the active site flap while the ortho nitro benzylidene moiety still stabilized in its initial position faced to Arg439 through the electrostatic interaction (Fig. 10b, down) (the types of residue interactions during the MD simulation represents in different color by Fig. 10c). So, it can propose that compound **7h** provides the stabilized interaction to Arg439 at the opening part of the active site through its polar nitro substitution. This finding is in accordance with our experimental results as the molecular level explanation on the higher urease activity of compound **7h** and the compounds with polar substituent at the phenyl ring. Also, again the E-ap conformer, as the preferred geometrical isomer by the benzylidene hydrazone, recognized for both stages of MD simulation time.

Furthermore, Fig. 10d shows the detailed 2D-ligand atom interactions that occurred more than 30.0% of the simulation time during the equilibrated phase over urease complexed with thiourea and compound **7h**. The interaction analysis suggests compound **7h** stabilized by the ortho nitro group with Arg439 at the entrance of active site through salt-bridge interaction for about 43% of simulation time. Also, it formed water-mediated H-bond interaction with Arg609 and Arg611 at on side of the active site flap through the C4 carbonyl group of barbituric acid ring for 45% and 55% of simulation time, respectively. In addition, Cys592 on the other side of the active site flap provided the non-bonding interaction mostly through hydrophobic interaction for about 45% of MD simulation time (Fig. 10c, Cys592 bar chart).

It is noteworthy that Arg609 and Cys592 are of the key residues at the root part of mobile flap covering the active site. Interacting with the mentioned residue for a significant amount of time, affects the flexibility of the mobile flap covering the active site and causes inhibition of the ureolytic activity^20^.

Comparing the results of compound **7h** interaction with thiourea indicates thiourea did not show any interaction with these key residues at the active site flap region which can proposed the reason of higher urease inhibition activity of compound **7h** rather than thiourea.

### In silico prediction of pharmacokinetic properties

The main physico-chemical properties of the synthesized compounds, which represent drug-likeness, partition coefficient, solubility, cell permeation, were calculated. Aiming to discuss the reliability of predictions in a consensus way, the parameters calculated with two different software. The partition coefficient (Log P) predicted by Qikpro module of schrodinger and swissADME web server while solubility (Log *S*), cell permeation and the predicted % human absorption (oral; %HOA and intestinal; %HIA) were measured based on Qikpro and pkCSM web server.

“Lipinski rule of five” was used to assess the drug-likeness of the synthesized compounds which includes calculating of the molecular weights, number of hydrogen bond donor, number of hydrogen bond acceptor and the predicted octanol/water partition co-efficient. According to Table 3, the number of violations of Lipinski’s rule of five (ROF violations) for all the synthesized compounds was zero (0) and therefore all compounds (**7a–o**) meet the drug-likeness criteria^26^.

**Table 3:** The calculated physico-chemical property of the synthesized compounds **7a–o** for predicting drug-likeness based on Lipinski-rule of five.

Compound	MW^a^	HBD^b^	HBA^c^	Log*P* o/w^d^		ROF violations^f^
				Qikpro	SwissADME (iLog P)	
**7a**	286.290	5	1	2.297	2.56	0
**7b**	302.289	2	5	1.373	1.72	0
**7c**	318.288	3	6	1.018	1.37	0
**7d**	376.368	1	7	2.354	3.02	0
**7e**	378.387	1	5	3.655	2.86	0
**7f**	320.735	1	4	2.627	2.55	0
**7g**	365.186	1	4	2.694	2.73	0
**7h**	331.287	1	6	1.412	1.6	0
**7i**	331.287	1	6	1.357	2.07	0
**7j**	355.180	1	4	2.993	2.49	0
**7k**	365.732	1	6	1.802	1.71	0
**7l**	365.732	1	6	1.916	1.90	0
**7m**	292.312	1	4	2.010	1.76	0
**7n**	326.757	1	4	2.522	2.4	0
**7o**	336.349	1	4	3.055	2.54	0
**Hydroxyurea**	76.06	3	2	− 0.2	− 0.16	0

^a^Molecular weights (acceptable value ≤ 500).

^b^Number of average Hydrogen Bond Donor (acceptable value ≤ 5).

^c^Number of average Hydrogen Bond Acceptor (acceptable value ≤ 10).

^d^Predicted octanol/water partition co-efficient (acceptable value ≤ 5).

^e^Number of violations of Lipinski’s rule of five (maximum 4).

Also, the bioavailability of a compound mainly depends on the absorption and metabolism process. The oral and gastrointestinal absorption procedures in turn relay on the solubility and permeability of the a compound^27^. The computed physico-chemical parameters used to assess absorption of the newly synthesized compounds **7a–o** and hydroxyurea including; the predicted aqueous solubility (Log *S*), the predicted apparent Caco-2 cell permeability as a model for the gut-blood barrier (non-active transport) (PCaco-2 and LogCaco-2), the predicted % human absorption (oral; %HOA and intestinal; %HIA) which were measured based on two different methods Qikpro and pkCSM web server and presented in Table 4. Comparing the experimental solubility value of hydroxyurea which considered as 100 mg/ml (experimental Log *S* of 1.2 mol/L) with the calculated Log *S* value of 1.1 (Table 4, Qikpro method) confirm the reliability of calculated data. Based on Jorgensen’s rule of three, orally availability is determined by calculating descriptor expressed as: log *S*_*wa*t_ >  − 5.7, PCaco-2 > 22 nm/s, and Primary Metabolites < 7^28^. According to Table 4, all the titled compounds follow of Jorgensen’s rule of three. Moreover, the same result has been revealed by implementing the pkCSM web server which proved the resulted outcome. In this way, compounds **7a**, **7d-g**, **7j**, and **7m–o** exhibited high HOA and the other compounds have moderate HOA.

**Table 4:** The calculated physico-chemical properties of the synthesized compounds **7a–o** for predicting absorption procedure.

No.	Qikpro					pkCSM		
	Log *S*_*wat*_^a^	PCaco-2^b^	% HOA^c^	metab^d^	RO3 V^e^	Log *S*	LogCaco-2f	%HIA^g^
**7a**	− 3.276	466.539	88.163	0	0	− 2.82	0.491	63.68
**7b**	− 3.452	52.356	67.24	2	0	− 2.73	− 0.051	54.28
**7c**	− 3.558	463.086	63.67	1	0	− 2.42	0.490	51.12
**7d**	− 3.750	466.982	88.503	3	0	− 3.42	0.315	60.12
**7e**	− 5.027	466.528	96.113	0	0	− 4.23	1.11	93.61
**7f**	− 4.043	466.707	90.100	0	0	− 3.52	0.50	67.59
**7g**	− 4.164	466.609	90.490	0	0	− 3.69	0.492	67.71
**7h**	− 3.326	61.598	67.243	1	0	− 3.43	− 0.184	62.36
**7i**	− 3.317	56.002	66.181	1	0	− 3.39	− 0.185	62.43
**7j**	− 4.539	469.380	92.283	0	0	− 4.21	0.874	92.05
**7k**	− 3.941	56.434	68.846	1	0	− 4.01	− 0.203	63.57
**7l**	− 4.088	61.624	70.194	1	0	− 4.01	− 0.20	63.60
**7m**	− 3.210	453.274	86.260	1	0	− 3.10	0.486	70.21
**7n**	− 3.997	453.958	89.268	1	0	− 3.83	0.493	74.30
**7o**	− 4.484	471.159	92.678	0	0	− 3.23	0.448	94.16
**Hu**	1.1	38.71	40.93	1	0	0.70	0.494	73.12

^a^Predicted aqueous solubility in mol dm^−3^ (− 6.5 to 0.5) (QPlogS > − 5.7).

^b^Predicted Caco-2 cell permeability in nm/s.

^c^Percentage human oral absorption (< 25% is poor and > 80% is high).

^d^Number of likely metabolic reactions (Primary Metabolites < 7).

^e^Number of violations of Jorgensen’s rule of three. Compounds with fewer (preferably no) violations of these rules are more likely to be orally available.

^f^Predicted Caco-2 cell permeability of a given compound is given as the log Papp in 10^−6^ cm/s.

^g^Percent of human intestinal absorption, (< 30% is poor and > 30% is high).

## Conclusion

In summary, a novel series of arylmethylene hydrazine-1,3-dimethylbarbituric derivatives **7a–o** have been synthesized via simple chemical reactions, and their inhibitory activities against urease were evaluated. In the enzymatic assay, all the synthesized compounds **7a–o** acted as potent inhibitors against urease (IC_50_ values = 0.61 ± 0.06–4.56 ± 0.18 μM) and were more active than the standard inhibitors hydroxyurea and thiourea (IC_50_ values = 100 ± 0.15 and 23 ± 1.7 μM, respectively). Furthermore, IFD study of the synthesized compounds in the urease active site showed that compounds with higher urease inhibitor activity could interact with higher number of residues, specially Arg609, Cys592 and showed higher computed free energy, while compounds with lower urease activity could not provide the proper interaction with Arg609, and Cys592 as the key interacting residues along with lower free binding energy. MD investigation revealed compound **7h** interacted with Arg609 and Cys592 which are of the key residues at the root part of mobile flap covering the active site. Interacting with the mentioned residue for a significant amount of time, affects the flexibility of the mobile flap covering the active site and causes inhibition of the ureolytic activity. Moreover, in silico pharmacokinetic study predicted that newly synthesized compounds are drug-likeness and can be orally active.

## Experimental

### Methods

Melting points of arylmethylene hydrazine-1,3-dimethylbarbituric derivatives **7a–o** were taken on a Kofler hot‐stage apparatus. ^1^H spectra were recorded on Bruker FT‐500 (500 MHz), and are reported relative to DMSO-*d*_*6*_ (δ 2.50). ^1^H NMR coupling constants (*J*) are reported in Hertz (Hz), and multiplicities are indicated as follows: s (singlet), d (doublet), t (triplet), m (multiplet), dd (doublet of doublet), dt (doublet of triplet). Proton-decoupled ^13^C NMR spectra were recorded on Bruker FT‐500 (125 MHz) and reported relative to DMSO-*d*_*6*_ (δ 40.0). The infrared (IR) spectra of title compounds were obtained on a Nicolet Magna FT‐IR 550 spectrophotometer (KBr disks). Elemental analysis of compounds **7a–o** was carried out with an Elementar Analysensysteme GmbH VarioEL CHN mode. Spectra data of arylmethylene hydrazine-1,3-dimethylbarbituric derivatives **7a–o** are available in the supplementary information.

### Synthesis of 5-(methoxymethylene)-1,3-dimethylbarbituric acid 3

A mixture of 1,3-dimethylbarbituric acid **1** (1 mmol) and trimethoxymethane **2** (3 mmol) in ethanol (5 ml) was refluxed for 3 h. Then, this mixture was allowed to stand overnight at room temperature for formation pure 5-(methoxymethylene)-1,3-dimethylbarbituric acid **3**.

### Synthesis of 5-(hydrazineylmethylene)-1,3-dimethylbarbituric acid 5

A mixture of 5-(methoxymethylene)-1,3-dimethylbarbituric acid **3** (1 mmol) and hydrazine **4** (1 mmol) in ethanol (5 ml) was refluxed for 1 h. After that, the reaction mixture was allowed to cool at room temperature and poured into water, and the pure 5-(hydrazineylmethylene)-1,3-dimethylbarbituric acid **5** were filtered off.

### General procedure for the synthesis of Arylmethylene hydrazine-1,3-dimethylbarbituric derivatives 7a–o

A mixture of the compound **5** (1 mmol), aromatic aldehydes **6a–o** (1.5 mmol), PTSA in ethanol (10 ml) was was stirred at room temperature for 8 h. The obtained precipitate was filtered off and washed with ethanol (2 ml) to give pure compounds **7a–o**.

**(E)-5-((2-benzylidenehydrazinyl)methylene)-1,3-dimethylpyrimidine-2,4,6(1H,3H,5H)-trione (7a).** White solid; isolated yield: 96%, mp 172–174 °C; IR (KBr, υ): 3289, 3057, 2929, 1638 cm^-1^; ^1^H NMR (301 MHz, DMSO-*d*_6_) δ 12.71 (s, 1H, NH), 8.78 (s, 1H, CH=N), 8.44 (s, 1H, CH=C), 7.76 (dd, *J* = 6.6, 2.8 Hz, 2H), 7.54 – 7.48 (m, 3H), 3.20 (s, 6H, 2CH_3_); ^13^C NMR (76 MHz, DMSO-*d*_6_) δ 159.96, 154.44, 151.91, 133.23, 131.74, 129.48, 128.21, 121.88, 117.10, 90.73, 28.06 (CH_3_), 27.46 (CH_3_); Anal Calcd for C_14_H_14_N_4_O_3_, C, 58.73, H, 4.93, N, 19.57 found: C, 58.70, H, 4.98, N, 19.50.

**(E)-5-((2-(2-hydroxybenzylidene)hydrazinyl)methylene)-1,3-dimethylpyrimidine-2,4,6(1H,3H,5H)-trione (7b).** White solid; isolated yield: 83%, mp 173–175 °C; IR (KBr, υ): 3284, 3057, 2939, 1631 cm^-1^; ^1^H NMR (301 MHz, DMSO-*d*_6_) δ 12.62 (d, *J* = 12.0 Hz, 1H, NH), 10.17 (s, 1H, OH), 8.79 (s, 1H, CH=N), 8.45 (d, *J* = 12.0 Hz, 1H, CH=C), 8.29 (d, *J* = 7.8 Hz, 1H, H^3^), 7.47 (m, 1H, H^4^), 6.44 – 6.31 (m, 2H, H^5^ & H^6^), 3.19 (s, 3H, CH_3_), 3.18 (s, 3H, CH_3_); ^13^C NMR (76 MHz, DMSO-*d*_6_) δ 163.40, 162.41, 159.78, 153.54, 152.64, 151.90, 129.92, 110.68, 108.72, 102.92, 90.43, 89.77, 28.00 (CH_3_), 27.39 (CH_3_); Anal Calcd for C_14_H_14_N_4_O_4_, C, 55.63, H, 4.67, N, 18.53 found: C, 55.65, H, 4.64, N, 18.48.

**(E)-5-((2-(2,4-dihydroxybenzylidene)hydrazinyl)methylene)-1,3-dimethylpyrimidine-2,4,6(1H,3H,5H)-trione (7c).** White solid; isolated yield: 80%, mp 184–186 °C; IR (KBr, υ): 3292, 3048, 2936, 1639 cm^-1^; ^1^H NMR (301 MHz, DMSO-*d*_6_) δ 12.64 (s, 1H, NH), 10.18 (s, 1H, OH), 10.09 (s, 1H, OH), 8.80 (s, 1H, CH=N), 8.46 (s, 1H, CH=C), 7.48 (d, *J* = 9.1 Hz, 1H, H^6^), 6.42–6.30 (m, 2H), 3.19 (s, 6H, 2CH_3_); ^13^C NMR (76 MHz, DMSO-*d*_6_) δ 163.41, 162.42, 162.27, 159.79, 153.58, 152.67, 151.95, 129.93, 110.72, 108.75, 102.95, 89.79, 28.02 (CH_3_), 27.43 (CH_3_); Anal Calcd for C_14_H_14_N_4_O_5_, C, 52.83, H, 4.43, N, 17.60 found: C, 52.88, H, 4.40, N, 17.67.

**(E)-1,3-dimethyl-5-((2-(3,4,5-trimethoxybenzylidene)hydrazinyl)methylene)pyrimidine-2,4,6(1H,3H,5H)-trione (7d).** White solid; isolated yield: 94%, mp 197–199 °C; IR (KBr, υ): 3294, 3047, 2931, 1626 cm^-1^; ^1^H NMR (301 MHz, DMSO-*d*_6_) δ 12.82 – 12.51 (m, 1H, NH), 8.65 (s, 1H, CH=N), 8.49 (s, 1H), 7.07 (s, 2H), 3.86 (s, 6H, 2CH_3_), 3.74 (s, 3H), 3.20 (s, 6H); ^13^C NMR (76 MHz, DMSO-*d*_6_) δ 164.63, 158.97, 155.50, 150.67, 146.14, 140.03, 126.53, 106.53, 103.88, 94.75, 61.30 (OCH3), 55.46 (OCH3), 27.91 (CH_3_), 26.53 (CH_3_); Anal Calcd for C_17_H_20_N_4_O_6_, C, 54.25, H, 5.36, N, 14.89 found: C, 54.28, H, 5.30, N, 14.88.

**(E)-1,3-dimethyl-5-((2-(3-phenoxybenzylidene)hydrazinyl)methylene)pyrimidine-2,4,6(1H,3H,5H)-trione (7e).** White solid; isolated yield: 87%, mp 217–219 °C; IR (KBr, υ): 3281, 3056, 2935, 1639 cm^-1^; ^1^H NMR (301 MHz, DMSO-*d*_6_) δ 12.68 (s, 1H, NH), 8.73 (s, 1H, CH=N), 8.35 (s, 1H, CH=C), 7.52 – 7.41 (m, 4H), 7.32 (s, 1H, H^6^), 7.21 (t, *J* = 7.4 Hz, 1H), 7.14 (dt, *J* = 7.2, 2.3 Hz, 1H), 7.08 (d, *J* = 7.6 Hz, 2H), 3.18 (s, 6H, 2CH_3_); ^13^C NMR (76 MHz, DMSO-*d*_6_) δ 157.77, 156.56, 154.46, 153.54, 151.85, 137.88, 135.16, 131.19, 130.66, 126.07, 124.44, 123.56, 121.67, 119.50, 116.88, 90.88, 27.50 (CH_3_), 26.08 (CH_3_); Anal Calcd for C_20_H_18_N_4_O_4_, C, 63.48, H, 4.79, N, 14.81 found: C, 63.41, H, 4.83, N, 14.86.

**(E)-5-((2-(4-chlorobenzylidene)hydrazinyl)methylene)-1,3-dimethylpyrimidine-2,4,6(1H,3H,5H)-trione (7f).** White solid; isolated yield: 92%, mp 179–181 °C; IR (KBr, υ): 3290, 3059, 2928, 1631 cm^-1^; ^1^H NMR (301 MHz, DMSO-*d*_6_) δ 12.73 (d, *J* = 11.9 Hz, 1H, NH), 8.78 (s, 1H, CH=N), 8.41 (d, *J* = 11.8 Hz, 1H, CH=C), 7.76 (d, *J* = 8.5 Hz, 2H, H^2^&H^6^), 7.57 (d, *J* = 8.5 Hz, 2H, H^3^&H^5^), 3.21 (s, 3H, CH_3_), 3.19 (s, 3H, CH_3_); ^13^C NMR (76 MHz, DMSO-*d*_6_) δ 159.50, 156.03, 153.92, 149.09, 144.03, 141.19, 137.84, 131.96, 129.32, 98.30, 27.89 (CH_3_), 25.25 (CH_3_); Anal Calcd for C_14_H_13_ClN_4_O_3_, C, 52.43, H, 4.09, N, 17.47 found: C, 52.46, H, 4.05, N, 17.52.

**(E)-5-((2-(3-bromobenzylidene)hydrazinyl)methylene)-1,3-dimethylpyrimidine-2,4,6(1H,3H,5H)-trione (7g).** White solid; isolated yield: 94%, mp 207–209 °C; IR (KBr, υ): 3293, 3055, 2932, 1637 cm^-1^; ^1^H NMR (301 MHz, DMSO-*d*_6_) δ 12.75 (s, 1H, NH), 8.73 (s, 1H, CH=N), 8.48 (s, 1H, CH=C), 7.94 (s, 1H, H^2^), 7.77–7.67 (m, 2H, H^4^&H^6^), 7.47 (t, *J* = 7.9 Hz, 1H, H^5^), 3.20 (s, 6H, 2CH_3_); ^13^C NMR (76 MHz, DMSO-*d*_6_) δ 154.44, 151.91, 149.42, 143.87, 138.34, 133.23, 131.75, 129.48, 128.21, 122.74, 121.79, 90.73, 27.07 (CH_3_), 26.78 (CH_3_); Anal Calcd for C_14_H_13_BrN_4_O_3_, C, 46.05, H, 3.59, N, 15.34 found: C, 46.09, H, 3.53, N, 15.30.

**(E)-1,3-dimethyl-5-((2-(2-nitrobenzylidene)hydrazinyl)methylene)pyrimidine-2,4,6(1H,3H,5H)-trione (7h).** White solid; isolated yield: 92%, mp 178–180 °C; IR (KBr, υ): 3291, 3048, 2928, 1638 cm^-1^; ^1^H NMR (301 MHz, DMSO-*d*_6_) δ 12.93 (s, 1H, NH), 9.19 (s, 1H, CH=N), 8.43 (d, *J* = 16.5 Hz, 1H, CH=C), 8.11 (s, 2H), 7.84 (s, 1H, H^5^), 7.74 (s, 1H, H^4^), 3.20 (s, 6H, 2CH_3_); ^13^C NMR (76 MHz, DMSO-*d*_6_) δ 163.30, 162.20, 153.82, 151.88, 148.91, 145.29, 140.42, 137.59, 133.56, 131.36, 128.75, 125.09, 90.60, 28.07 (CH_3_), 27.45 (CH_3_); Anal Calcd for C_14_H_13_N_5_O_6_, C, 48.42, H, 3.77, N, 20.17 found: C, 48.40, H, 3.70, N, 20.20.

**(E)-1,3-dimethyl-5-((2-(4-nitrobenzylidene)hydrazinyl)methylene)pyrimidine-2,4,6(1H,3H,5H)-trione (7i).** White solid; isolated yield: 86%, mp 171–173 °C; IR (KBr, υ): 3282, 3047, 2934, 1641 cm^-1^; ^1^H NMR (301 MHz, DMSO-*d*_6_) δ 12.83 (s, 1H, NH), 8.89 (s, 1H, CH=N), 8.47 (s, 1H, CH=C), 8.33 (d, *J* = 8.6 Hz, 2H, H^3^&H^5^), 8.00 (d, *J* = 8.7 Hz, 2H, H^3^&H^5^), 3.21 (s, 6H, 2CH_3_); ^13^C NMR (76 MHz, DMSO-*d*_6_) δ 162.97, 156.56, 153.92, 149.09, 143.50, 142.14, 138.90, 130.07, 125.85, 96.11, 28.95 (CH_3_), 27.36 (CH_3_); Anal Calcd for C_14_H_13_N_5_O_5_, C, 50.76, H, 3.96, N, 21.14 found: C, 50.79, H, 3.90, N, 21.20.

**(E)-5-((2-(2,3-dichlorobenzylidene)hydrazinyl)methylene)-1,3-dimethylpyrimidine-2,4,6(1H,3H,5H)-trione (7j).** White solid; isolated yield: 81%, mp 201–203 °C; IR (KBr, υ): 3296, 3051, 2935, 1635 cm^−1^; ^1^H NMR (301 MHz, DMSO-*d*_6_) δ 12.93 (d, *J* = 11.7 Hz, 1H, NH), 9.23 (s, 1H, CH=N), 8.38 (d, *J* = 11.6 Hz, 1H, CH=C), 7.96 (d, *J* = 7.9 Hz, 1H, H^4^), 7.76 (d, *J* = 8.0 Hz, 1H, H^6^), 7.45 (t, *J* = 8.0 Hz, 1H, H^5^), 3.18 (s, 6H, 2CH_3_); ^13^C NMR (76 MHz, DMSO-*d*_6_) δ 162.76, 160.22, 155.90, 152.72, 146.60, 143.80, 140.40, 135.48, 128.84, 127.02, 124.72, 124.38, 90.45, 27.78 (CH_3_), 25.79 (CH_3_); Anal Calcd for C_14_H_12_Cl_2_N_4_O_3_, C, 47.34, H, 3.41, N, 15.77 found: C, 47.30, H, 3.45, N, 15.70.

**(E)-5-((2-(2-chloro-5-nitrobenzylidene)hydrazinyl)methylene)-1,3-dimethylpyrimidine 2,4,6(1H,3H,5H)-trione (7k).** White solid; isolated yield: 83%, mp 188–190 °C; IR (KBr, υ): 3285, 3052, 2927, 1633 cm^-1^; ^1^H NMR (301 MHz, DMSO-*d*_6_) δ 13.02 (s, 1H, NH), 9.25 (s, 1H, CH=N), 8.70 (s, 1H, H^6^), 8.50 (s, 1H, H^4^), 8.29 (d, *J* = 11.7 Hz, 1H, CH=C), 7.87 (d, *J* = 8.9 Hz, 1H), 3.20 (s, 6H, 2CH_3_); ^13^C NMR (76 MHz, DMSO-*d*_6_) δ 161.84, 157.69, 154.60, 153.61, 148.63, 144.86, 136.33, 133.08, 131.05, 128.49, 126.00, 125.39, 93.02, 25.80 (CH_3_), 24.88 (CH_3_). Anal Calcd for C_14_H_12_ClN_5_O_5_, C, 45.98, H, 3.31, N, 19.15 found: C, 45.96, H, 3.35, N, 19.13.

**(E)-5-((2-(5-chloro-2-nitrobenzylidene)hydrazinyl)methylene)-1,3-dimethylpyrimidine-2,4,6(1H,3H,5H)-trione (7l).** White solid; isolated yield: 88%, mp 191–193 °C; IR (KBr, υ): 3286, 3053, 2934, 1630 cm^-1^; ^1^H NMR (301 MHz, DMSO-*d*_6_) δ 13.01 (s, 1H, NH), 9.17 (s, 1H, CH=N), 8.51 (s, 1H, H^6^), 8.18–8.13 (m, 2H), 7.84–7.78 (m, 1H, H^4^), 3.21 (s, 6H, 2CH_3_); ^13^C NMR (76 MHz, DMSO-*d*_6_) δ 163.82, 161.17, 159.79, 154.89, 153.82, 147.26, 143.33, 140.07, 134.59, 126.90, 123.77, 122.91, 93.08, 26.30 (CH_3_), 24.57 (CH_3_); Anal Calcd for C_14_H_12_ClN_5_O_5_, C, 45.98, H, 3.31, N, 19.15 found: C, 45.96, H, 3.38, N, 19.21.

**(E)-1,3-dimethyl-5-((2-(thiophen-2-ylmethylene)hydrazinyl)methylene)pyrimidine2,4,6(1H,3H,5H)-trione (7m).** White solid; isolated yield: 81%, mp 164–166 °C; IR (KBr, υ): 3288, 3052, 2930, 1635 cm^-1^; ^1^H NMR (301 MHz, DMSO-*d*_6_) δ 12.70 (s, 1H, NH), 8.97 (s, 1H, CH=N), 8.30 (s, 1H, CH=C), 7.81 (d, *J* = 5.0 Hz, 1H, H^5^), 7.56–7.52 (m, 1H, H^3^), 7.20 (dd, *J* = 5.0, 3.7 Hz, 1H, H^4^), 3.19 (s, 6H, 2CH_3_); ^13^C NMR (76 MHz, DMSO-*d*_6_) δ 163.30, 162.20, 153.82, 151.88, 148.90, 137.59, 133.56, 131.36, 128.75, 90.60, 28.07 (CH_3_), 27.45 (CH_3_); Anal Calcd for C_12_H_12_N_4_O_3_S, C, 49.31, H, 4.14, N, 19.17 found: C, 49.28, H, 4.17, N, 19.24.

**(E)-5-((2-((5-chlorothiophen-2-yl)methylene)hydrazinyl)methylene)-1,3-dimethylpyrimidine-2,4,6(1H,3H,5H)-trione (7n).** White solid; isolated yield: 88%, mp 185–187 °C; IR (KBr, υ): 3293, 3056, 2935, 1638 cm^-1^; ^1^H NMR (301 MHz, DMSO-*d*_6_) δ 12.93–12.48 (m, 1H, NH), 8.85 (s, 1H, CH=N), 8.37–8.30 (m, 1H, CH=C), 7.47–7.38 (m, 1H, H^4^), 7.27–7.19 (m, 1H, H^3^), 3.20 (s, 6H, 2CH_3_); ^13^C NMR (76 MHz, DMSO-*d*_6_) δ 162.25, 158.07, 153.90, 151.73, 148.03, 136.80, 133.36, 128.75, 124.66, 90.93, 28.12 (CH_3_), 27.48 (CH_3_); Anal Calcd for C_12_H_11_ClN_4_O_3_S, C, 44.11, H, 3.39, N, 17.15 found: C, 44.17, H, 3.42, N, 17.19.

**(E)-1,3-dimethyl-5-((2-(naphthalen-1-ylmethylene)hydrazinyl)methylene)pyrimidine-2,4,6(1H,3H,5H)-trione (7o).** White solid; isolated yield: 90%, mp 213–115 °C; IR (KBr, υ): 3292, 3057, 2933, 1639 cm^−1^; ^1^H NMR (301 MHz, DMSO-*d*_6_) δ 12.82 (s, 1H, NH), 9.53 (s, 1H, CH=N), 8.76 (d, *J* = 8.4 Hz, 1H, H^2^), 8.58 (s, 1H, CH=C), 8.12–8.02 (m, 3H), 7.73–7.60 (m, 3H), 3.20 (s, 6H, 2CH_3_); ^13^C NMR (76 MHz, DMSO-*d*_6_) δ 163.43, 155.47, 147.22, 143.86, 135.72, 133.54, 131.66, 130.81, 129.40, 127.03, 124.78, 123.21, 121.65, 117.64, 97.80, 26.21 (CH_3_), 24.66 (CH_3_); Anal Calcd for C_18_H_16_N_4_O_3_, C, 64.28, H, 4.79, N, 16.66 found: C, 64.33, H, 4.72, N, 16.60.

### Urease inhibitory activity and kinetic study

All used material and *JB* urease (EC 3.5.1.5) were purchased from Sigma–Aldrich (USA). Potassium phosphate buffer (PPB) solution with concentration 100 mM and pH = 7.4 was prepared in distilled water. Urease inhibition effects of the synthesized compounds **7a–o** was determined using the modified Berthelot spectrophotometric method by a Synergy H1 Hybrid multi-mode microplate reader (BioTek, Winooski, VT, USA) at 625 nm^16–20^. The enzymatic reactions were performed in PPB solution which reach to the 985 μL by adding urea (850 μL) and the synthesized compound (100 μL, 0–10 mg/mL). Then, urease (15 μL) was added and the concentration of liberated ammonia was measured after 60 min. The corresponding concentration of ammonia was determined by addition of the incubated solution (100 μL) to the mixture of 500 μL of solution I (5.0 g phenol and 25.0 mg sodium nitroprusside in 500 mL water) and 500 μL of solution II (2.5 g sodium hydroxide and 4.2 mL sodium hypochlorite (5% chlorine) in 500 mL water) which was incubated at 37 °C for 30 min. The absorbance was obtained by measuring indophenols. The activity of uninhibited *JB* urease was considered as the control activity of 100%. The inhibition assays were conducted according to this formula: I (%) = [1 − (T/C)] × 100; where I (%) is the enzyme inhibition, T (test) is the absorbance of the analyzed compounds in the presence of urease solution, and C (control) is the absorbance of the solvent in the presence of urease solution. Data were expressed as mean ± standard error (SD) and run in triplicate. The IC_50_ values for the all compounds **7a–o** were calculated using GraphPad Prism 5 software (GraphPad Software, Inc., San Diego, CA). Thiourea and hydroxyurea were used as the standard inhibitors for urease. For the kinetic study the urea concentrations were changed from 3.12 to 100 mM and concentrations 0, 0.5, and 1 µM of the most potent urease inhibitor was used.

### Molecular modeling procedure

In order to find out the interactions mode of designed molecules over urease enzyme, Maestro Molecular Modeling platform (version10.5) by Schrödinger, LLC was performed^29^. The X-ray crystallographic structure of *Jack bean* urease (*JB* urease) (in complex with acetohydroxamic acid, AHA) was downloaded from the Protein Data Bank (PDB ID; 4h9m) (www.rcsb.org). As urease is reported to be functionally active in monomeric state, all the docking studies were performed on single monomer. In addition, prosthetic group and co-factors are not directly involved in urease inhibition, so they totally removed before docking investigation. Water molecules and co-crystallized ligands were removed from the enzymes crystallographic structures. The 2D structures of all synthesized compounds were drawn in Marvin 15.10.12.0 program (http://www.chemaxon.com) and converted into pdb file^30^. The Protein Preparation Wizard and the LigPrep module were used to prepare protein and ligand structure properly^31,32^. The missing side chains of the proteins were filled using the Prime tool and missing residues were updated.

The accurate side‑chain, backbone conformational changes or both during ligand binding at the active site of urease enzyme were predicted by IFD method using Glide software (Schrödinger LLC 2018, USA)^33^. The AHA binding site was used to generate the grid for IFD calculation. The maximum 20 poses with receptor and ligand van der Waals radii of 0.7 and 0.5, respectively considered. Residues within 5 Å of the AHA at the active site were refined followed by side-chain optimization. Structures whose Prime energy is more than 30 kcal/mol are eliminated based on extra precious Glide docking.

The ligand binding energies (ΔG bind) were calculated for each synthesized compound using Molecular mechanics/generalized born surface area (MM‑GBSA) modules (Schrödinger LLC 2018) based on the following equation;

$$ \Delta {\text{G}}_{Bind} = {\text{ E}}_{Complex} {-} \, \left[ {{\text{E}}_{{{\text{Re}} ceptor}} + {\text{E}}_{Ligand} } \right] $$

where ΔG _*Bind*_ is the calculated relative free energy which includes both ligand and receptor strain energy^33^. E_*Complex*_ is the MM/GBSA energy of the minimized complex, and E_*Ligand*_ is the MM/ GBSA energy of the ligand after removing it from the complex and allowing it to relax. E_*Receptor*_ is the MM-GBSA energy of relaxed protein after separating it from the ligand. The MM-GBSA calculation was performed based on the best pose structure obtained from IFD complexes.

### Molecular dynamic simulation

Molecular simulations of this study were performed using the Desmond v5.3 using Maestro interface (from Schrödinger 2018‐4 suite)^34^. The appropriate pose for MD simulation procedure of the compound was achieved by IFD method.

In order to build the system for MD simulation, the protein–ligand complexes were solvated with SPC explicit water molecules and placed in the center of an orthorhombic box of appropriate size in the periodic boundary condition. Sufficient counter‐ions and a 0.15 M solution of NaCl were also utilized to neutralize the system and to simulate the real cellular ionic concentrations, respectively. The MD protocol involved minimization, pre-production, and finally production MD simulation steps. In the minimization procedure, the entire system was allowed to relax for 2500 steps by the steepest descent approach. Then the temperature of the system was raised from 0 to 300 K with a small force constant on the enzyme in order to restrict any drastic changes. MD simulations were performed via NPT (constant number of atoms, constant pressure i.e. 1.01325 bar and constant temperature i.e. 300 K) ensemble. The Nose‐Hoover chain method was used as the default thermostat with 1.0 ps interval and Martyna‐Tobias‐Klein as the default barostat with 2.0 ps interval by applying isotropic coupling style. Long‐range electrostatic forces were calculated based on particle‐mesh‐based ewald approach with the he cut‐off radius for columbic forces set to 9.0 Å. Finally, the system subjected to produce MD simulations for 20 ns for each protein–ligand complex. During the simulation every 1000 ps of the actual frame was stored. The dynamic behavior and structural changes of the systems were analyzed by the calculation of the root mean square deviation (RMSD) and RMSF. Subsequently, the representative frames of the simulation extracted based on the clustering method from the equilibrated trajectory system for investigating of ligand–protein complex interaction.

### In silico pharmacokinetic properties of synthesized compounds

QikProp module of Schrodinger^35^, swissADME^36^, and pkCSM^37^ were used to calculate the important physico-chemical properties of the synthesized compounds like drug-likeness, partition coefficient, solubility, cell permeation.

## Supplementary Information

Supplementary Information.
