# Supplementary material for: Arylmethylene hydrazine derivatives containing 1,3-dimethylbarbituric moiety as novel urease inhibitors
Source: Sci Rep. 2021 May 19;11:10607. doi: 10.1038/s41598-021-90104-x (PMC8134453; doi:10.1038/s41598-021-90104-x)

**Support information**

**Arylmethylene Hydrazine Derivatives Containing 1,3-Dimethylbarbituric Moiety as Novel Urease Inhibitors**

Keyvan Pedrood^1^, Homa Azizian^2^, Mohammad Nazari Montazer^3^, Maryam Mohammadi‐Khanaposhtani^4^, Mohammad Sadegh Asgari^5^, Mehdi Asadi^3^, Saeed Bahadorikhalili^5^, Hossein Rastegar^6^, Bagher Larijani^1^, Massoud Amanlou^3^, Mohammad Mahdavi^1✉^

^1^Endocrinology and Metabolism Research Center, Endocrinology and Metabolism Clinical Sciences Institute, Tehran University of Medical Sciences, Tehran, Iran. ^✉^e-mail: momahdavi@sina.tums.ac.ir (M. Mahdavi). ^2^Department of Medicinal Chemistry, School of Pharmacy‐International Campus, Iran University of Medical Sciences, Tehran, Iran. ^3^Department of Medicinal Chemistry, Faculty of Pharmacy and Pharmaceutical Sciences Research Center, Tehran University of Medical Sciences, Tehran, Iran. ^4^Cellular and Molecular Biology Research Center, Health Research Institute, Babol University of Medical Sciences, Babol, Iran. ^5^School of Chemistry, College of Science, University of Tehran, Tehran, Iran. ^6^Cosmetic products research center, Iranian food and drug administration, MOHE, Tehran, Iran.

*5-((2-benzylidenehydrazinyl)methylene)-1,3-dimethylpyrimidine-2,4,6(1H,3H,5H)-trione* ***(7a)***

*
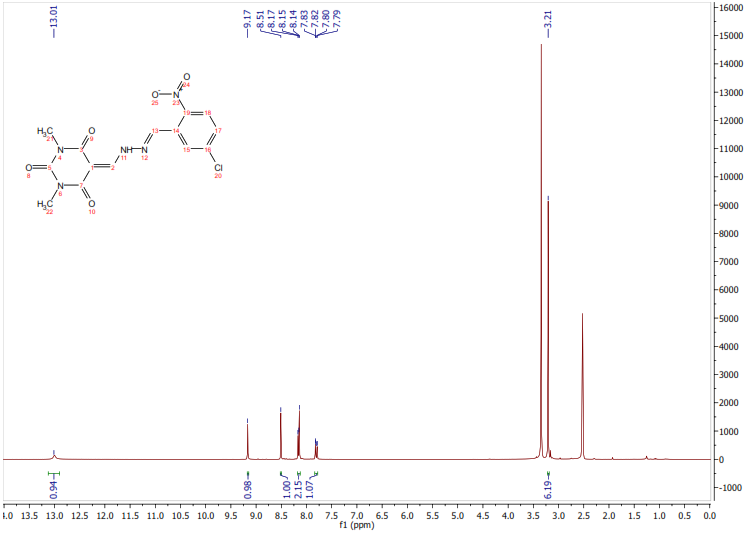
*

*
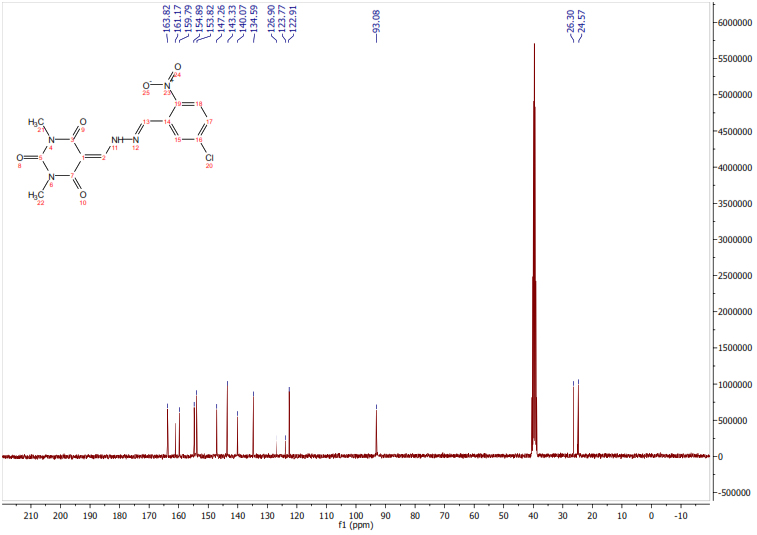
*

*5-((2-(2-hydroxybenzylidene)hydrazinyl)methylene)-1,3-dimethylpyrimidine-2,4,6(1H,3H,5H)-trione* ***(7b)***


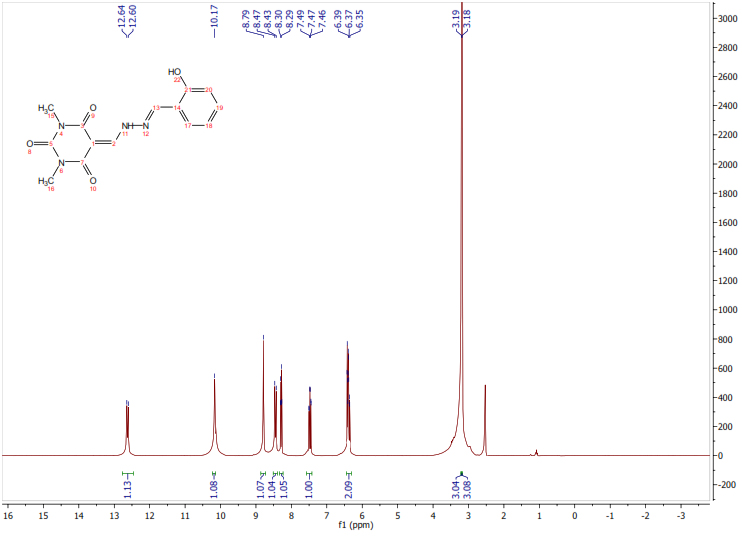


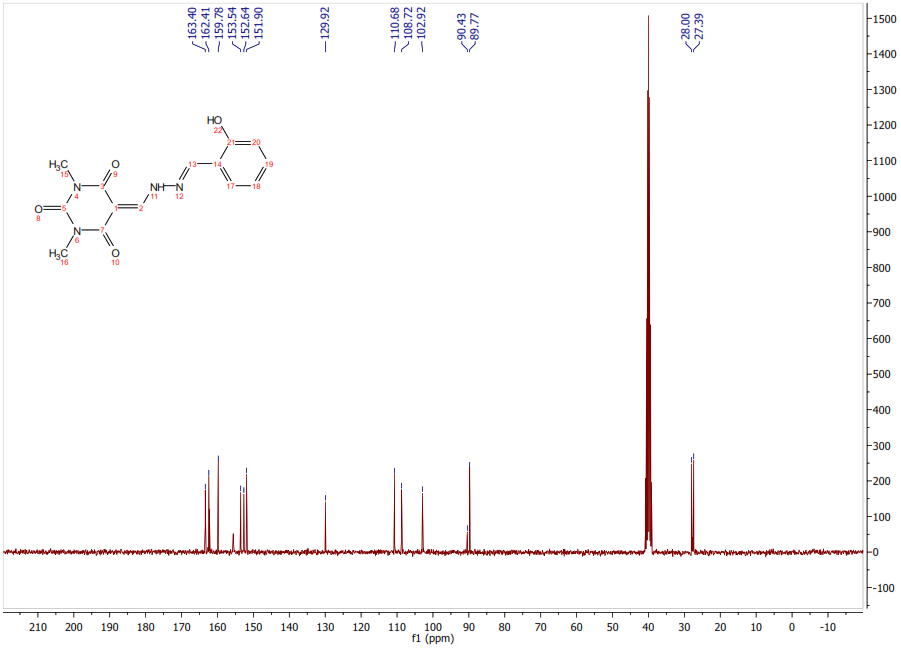


*5-((2-(2,4-dihydroxybenzylidene)hydrazinyl)methylene)-1,3-dimethylpyrimidine-2,4,6(1H,3H,5H)-trione* ***(7c)***


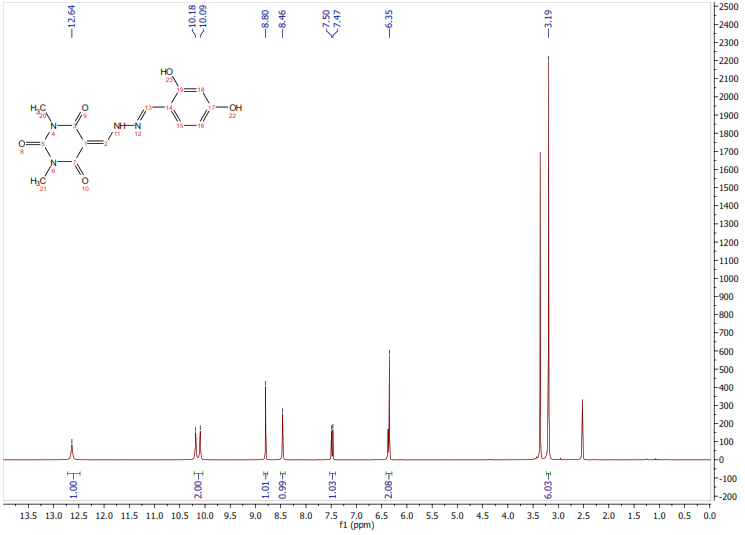


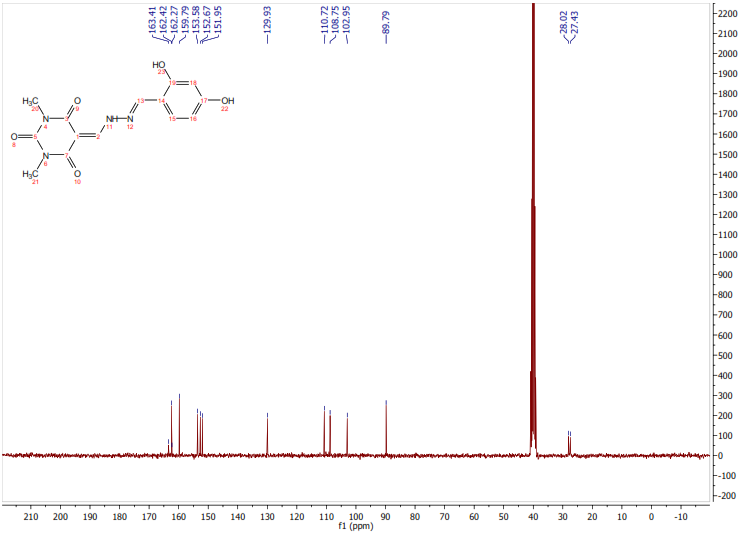


*1,3-dimethyl-5-((2-(3,4,5-trimethoxybenzylidene)hydrazinyl)methylene)pyrimidine-2,4,6(1H,3H,5H)-trione* ***(7d)***


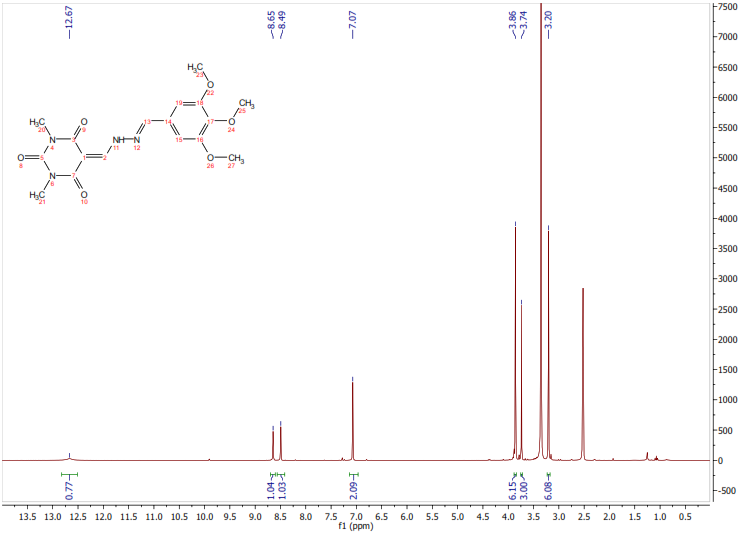


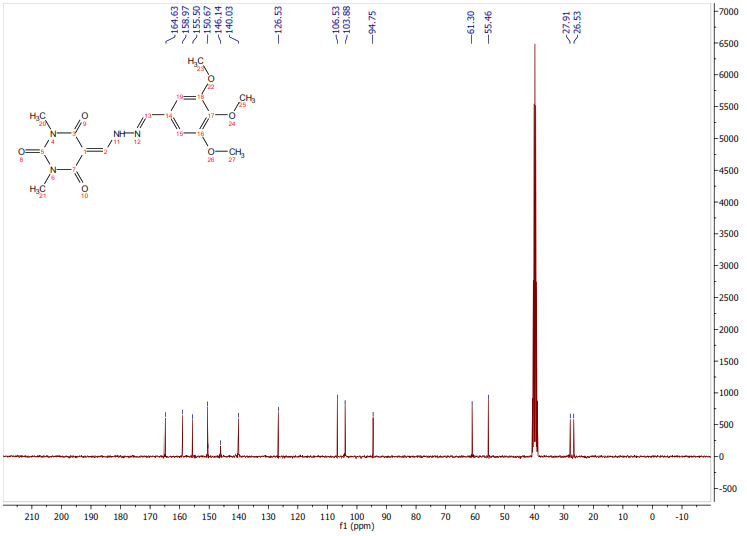


*1,3-dimethyl-5-((2-(3-phenoxybenzylidene)hydrazinyl)methylene)pyrimidine-2,4,6(1H,3H,5H)-trione* ***(7e)***


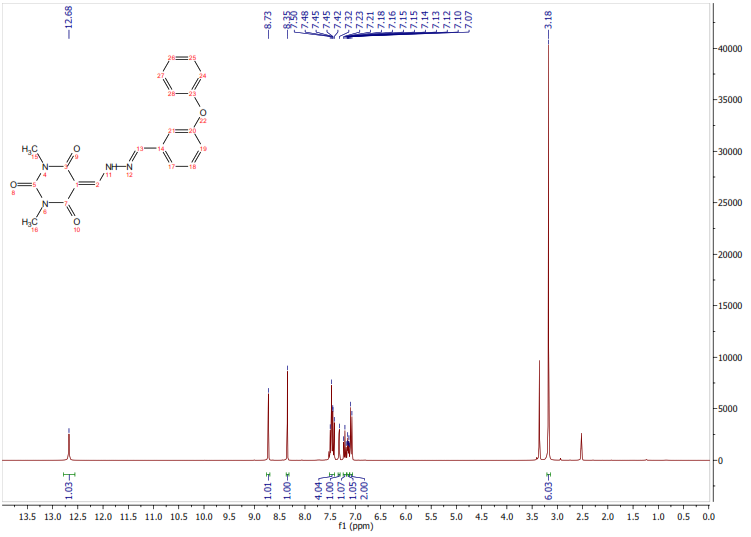


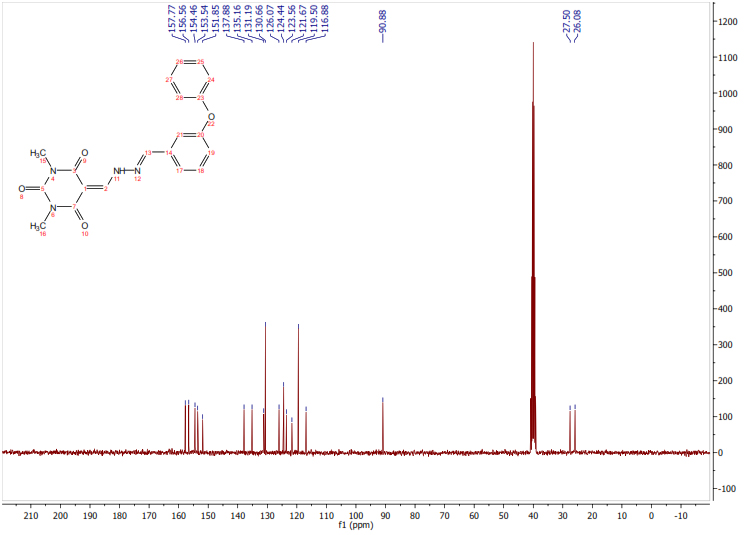


*5-((2-(4-chlorobenzylidene)hydrazinyl)methylene)-1,3-dimethylpyrimidine-2,4,6(1H,3H,5H)-trione* ***(7f)***


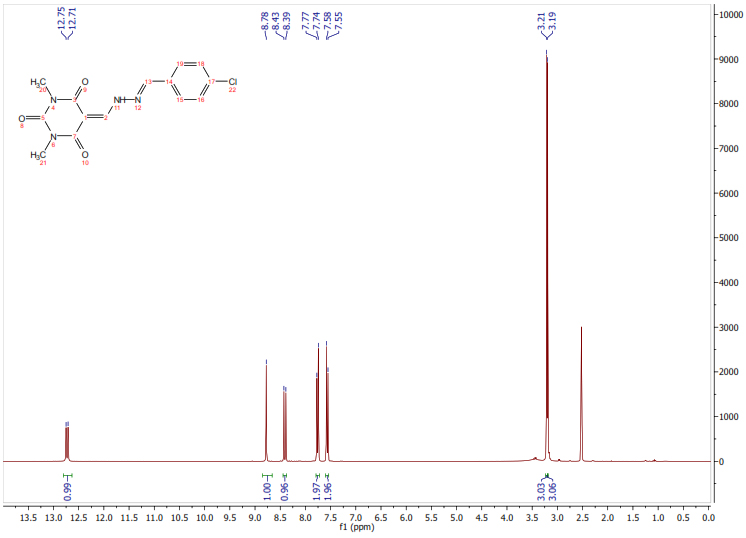


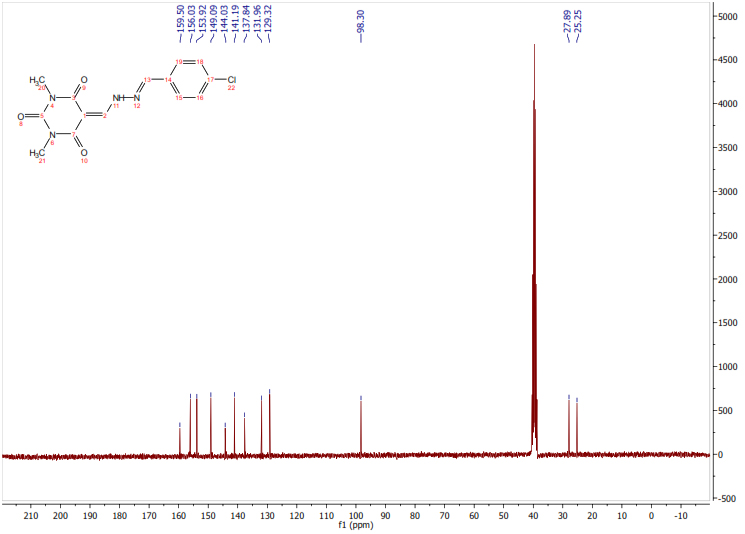


*5-((2-(3-bromobenzylidene)hydrazinyl)methylene)-1,3-dimethylpyrimidine-2,4,6(1H,3H,5H)-trione* ***(7g)***


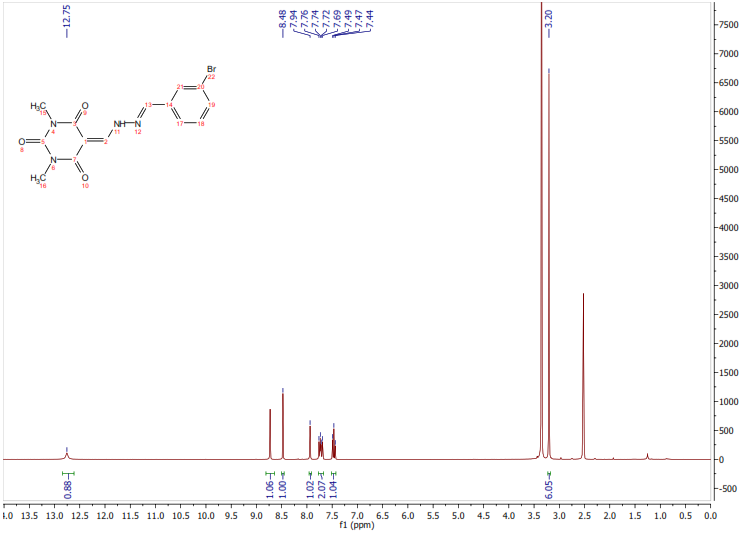


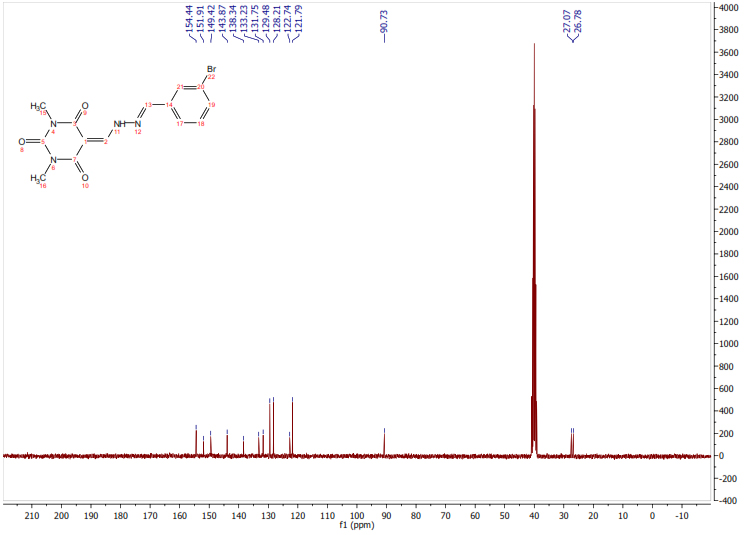


*1,3-dimethyl-5-((2-(2-nitrobenzylidene)hydrazinyl)methylene)pyrimidine-2,4,6(1H,3H,5H)-trione* ***(7h)***


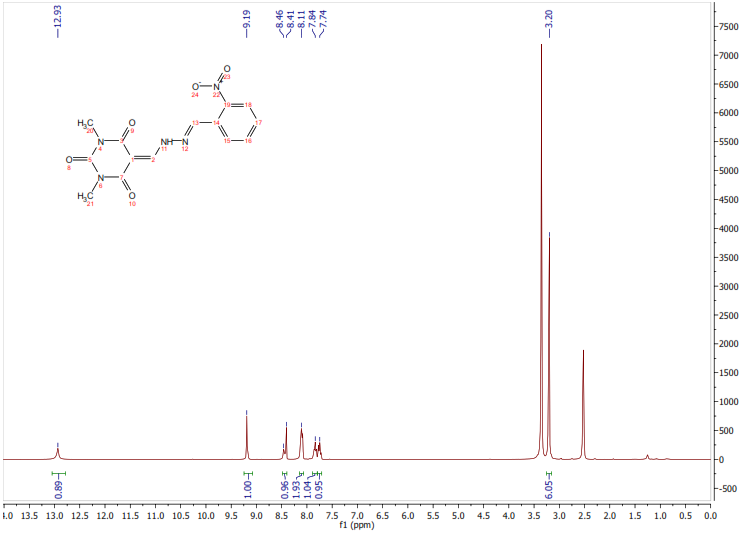


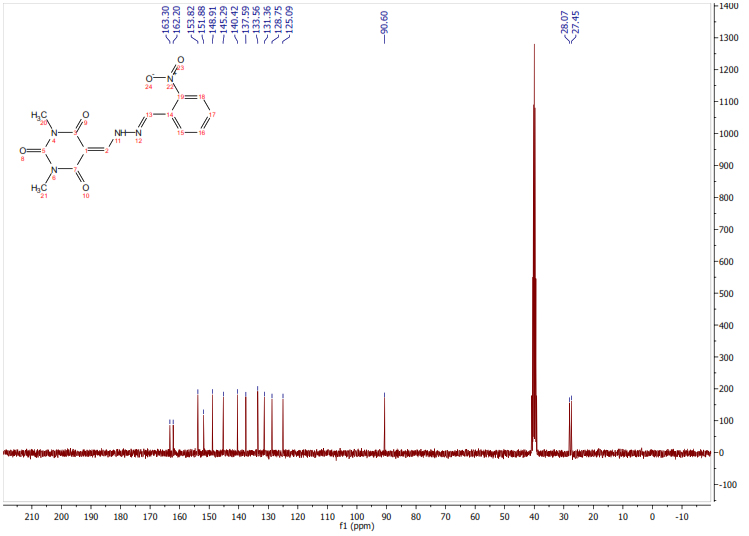


*1,3-dimethyl-5-((2-(4-nitrobenzylidene)hydrazinyl)methylene)pyrimidine-2,4,6(1H,3H,5H)-trione* ***(7i)***


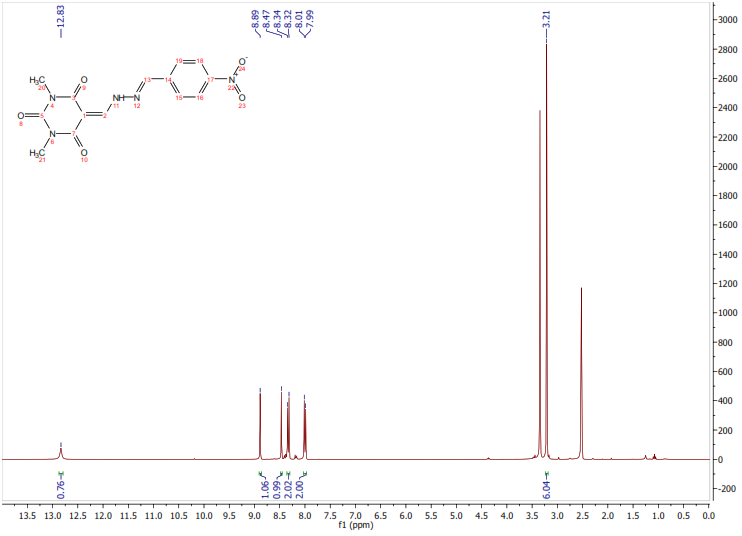

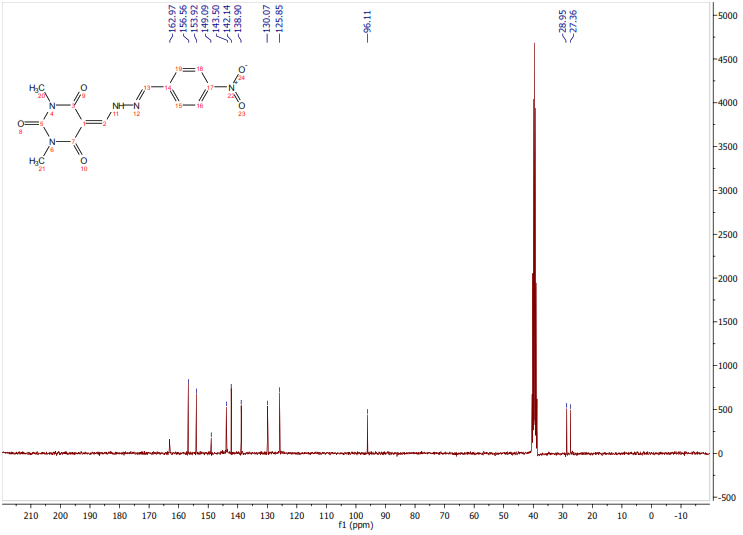


*5-((2-(2,3-dichlorobenzylidene)hydrazinyl)methylene)-1,3-dimethylpyrimidine-2,4,6(1H,3H,5H)-trione* ***(7j)***


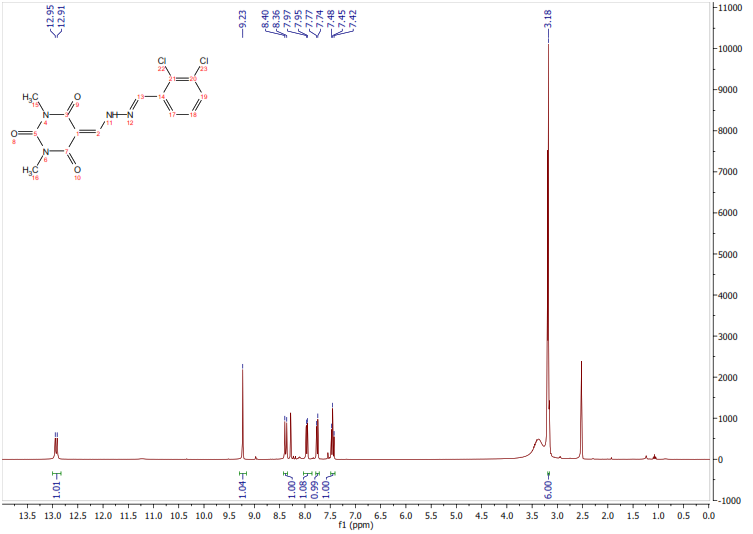


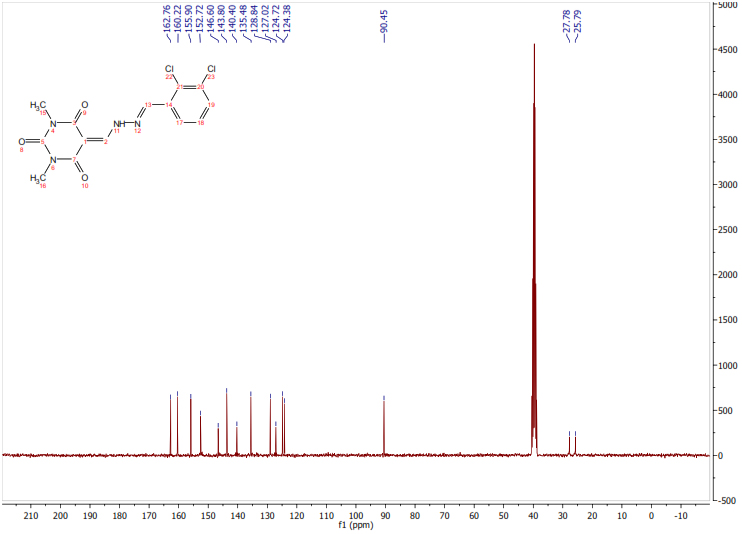


*5-((2-(2-chloro-5-nitrobenzylidene)hydrazinyl)methylene)-1,3-dimethylpyrimidine 2,4,6(1H,3H,5H)-trione* ***(7k)***


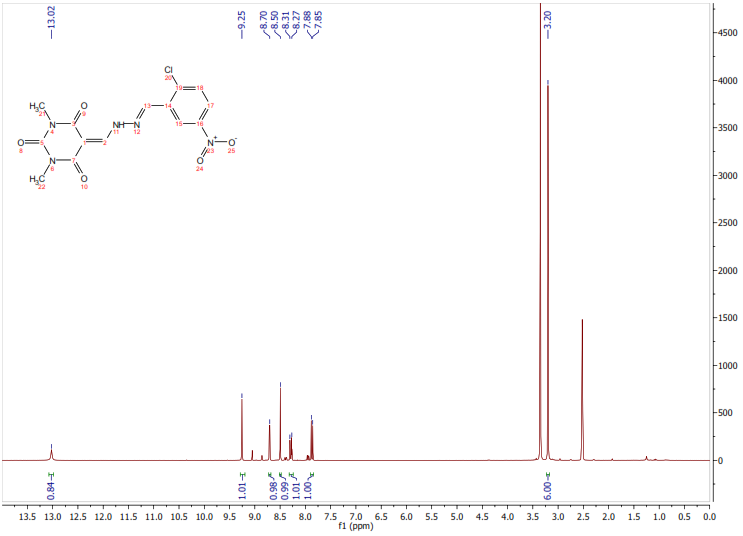


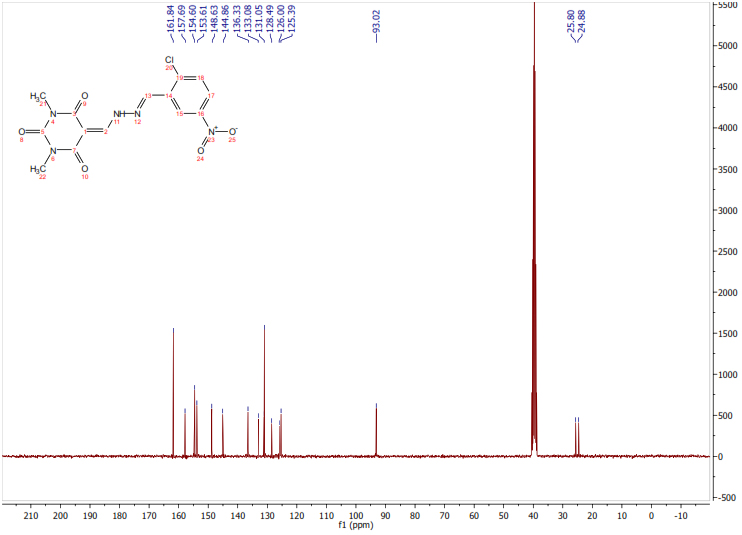


*5-((2-(5-chloro-2-nitrobenzylidene)hydrazinyl)methylene)-1,3-dimethylpyrimidine-2,4,6(1H,3H,5H)-trione* ***(7l)***


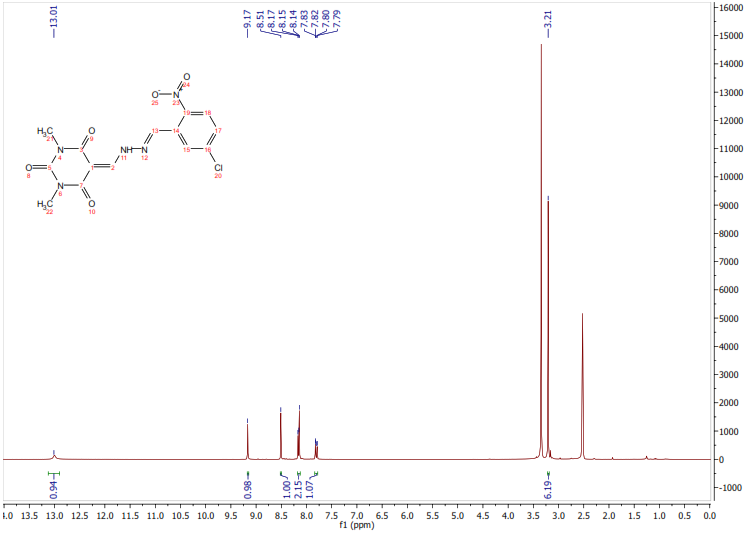


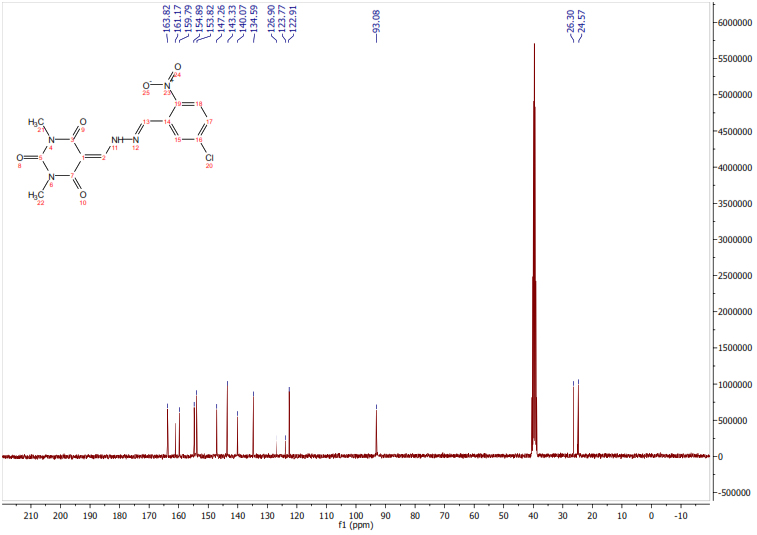


*1,3-dimethyl-5-((2-(thiophen-2-ylmethylene)hydrazinyl)methylene)pyrimidine2,4,6(1H,3H,5H)-trione* ***(7m)***


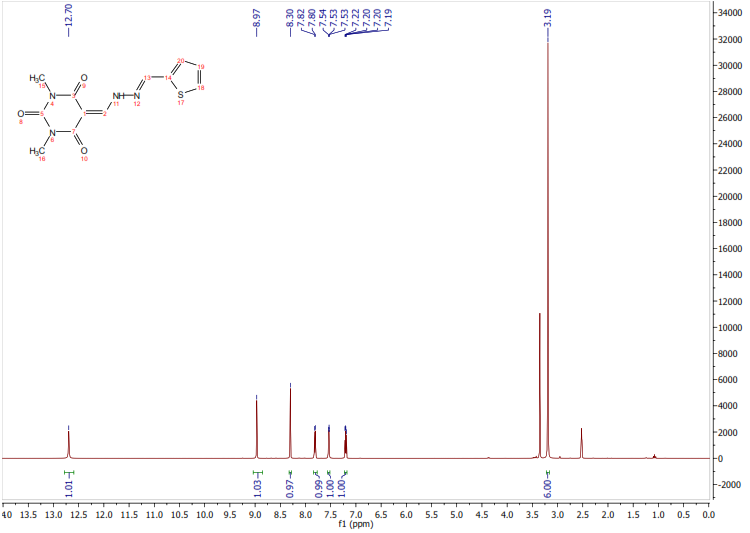


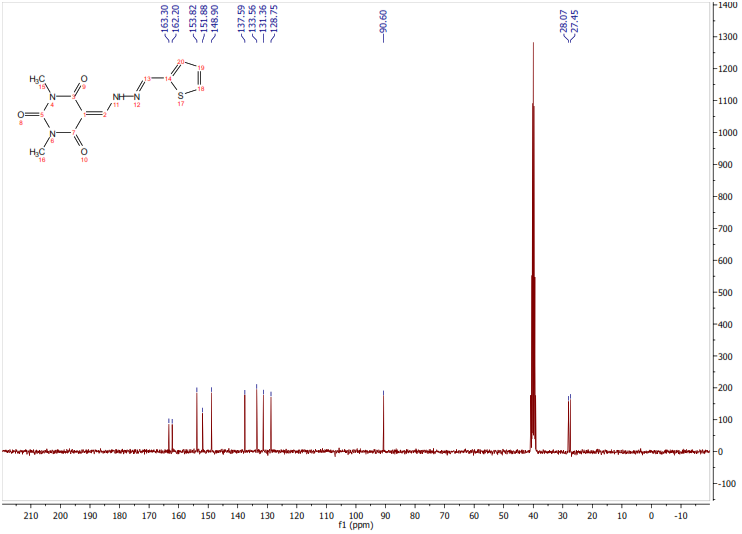


*5-((2-((5-chlorothiophen-2-yl)methylene)hydrazinyl)methylene)-1,3-dimethylpyrimidine-2,4,6(1H,3H,5H)-trione (****7n****)*


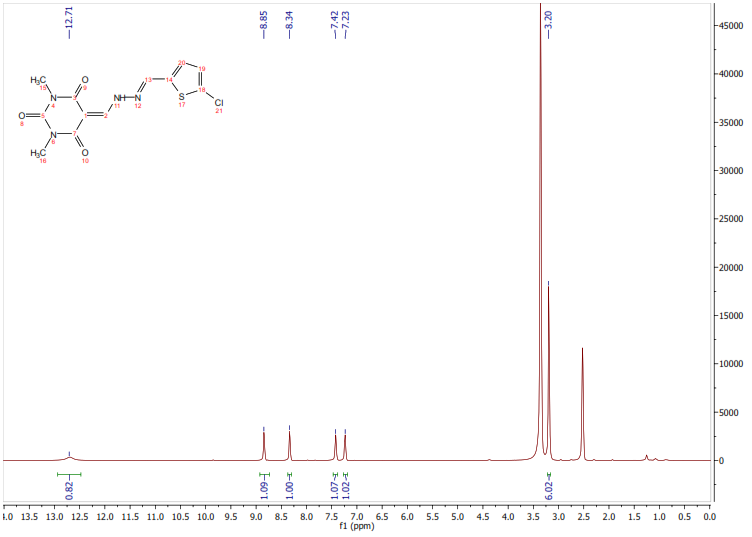


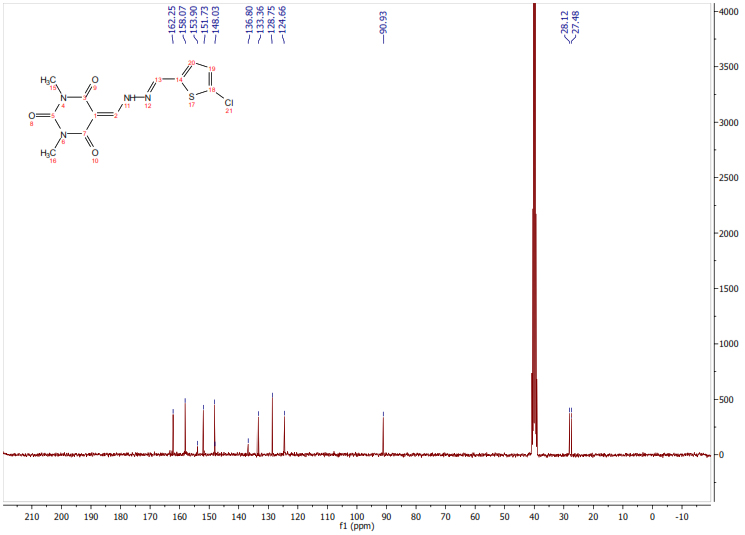


*(E)-1,3-dimethyl-5-((2-(naphthalen-1-ylmethylene)hydrazinyl)methylene)pyrimidine-2,4,6(1H,3H,5H)-trione* ***(7o)***


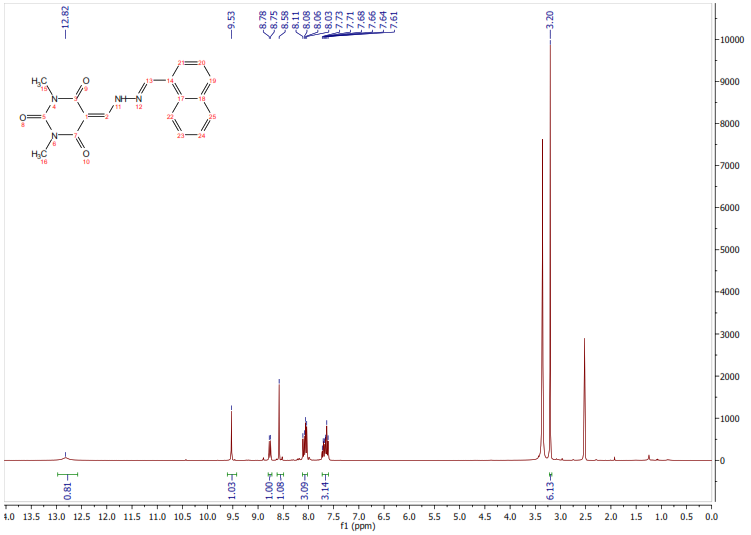


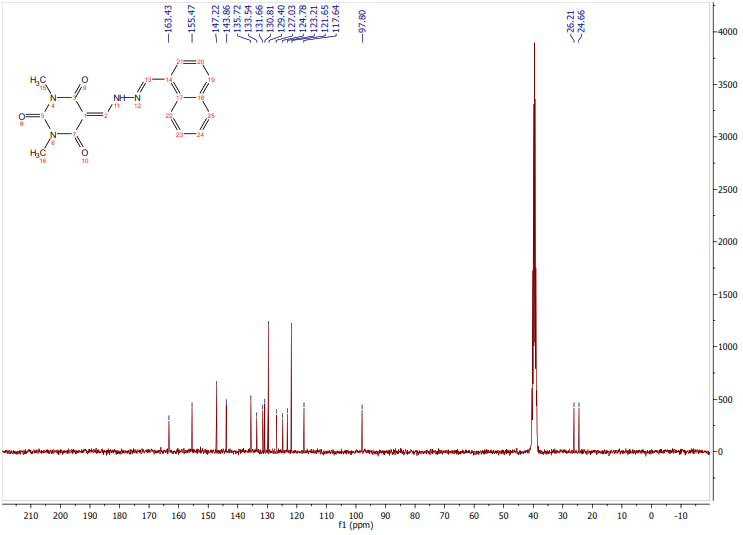

Supplement: Supplementary file 1 — Supplementary Information. [file 41598_2021_90104_MOESM1_ESM.docx]
